# Methionine Sulfoxide Reductase A (MsrA) and Its Function in Ubiquitin-Like Protein Modification in *Archaea*

**DOI:** 10.1128/mBio.01169-17

**Published:** 2017-09-05

**Authors:** Xian Fu, Zachary Adams, Rui Liu, Nathaniel L. Hepowit, Yifei Wu, Connor F. Bowmann, Jackob Moskovitz, Julie A. Maupin-Furlow

**Affiliations:** a Department of Microbiology and Cell Science, Institute of Food and Agricultural Sciences, University of Florida, Gainesville, Florida, USA; b Genetics Institute, University of Florida, Gainesville, Florida, USA; c Department of Pharmacology and Toxicology, University of Kansas, Lawrence, Kansas, USA; National Cancer Institute

**Keywords:** archaea, methionine sulfoxide reductase, oxidative stress, posttranslational modification, protein repair, ubiquitination

## Abstract

Methionine sulfoxide reductase A (MsrA) is an antioxidant enzyme found in all domains of life that catalyzes the reduction of methionine-*S*-sulfoxide (MSO) to methionine in proteins and free amino acids. We demonstrate that archaeal MsrA has a ubiquitin-like (Ubl) protein modification activity that is distinct from its stereospecific reduction of MSO residues. MsrA catalyzes this Ubl modification activity, with the Ubl-activating E1 UbaA, in the presence of the mild oxidant dimethyl sulfoxide (DMSO) and in the absence of reductant. In contrast, the MSO reductase activity of MsrA is inhibited by DMSO and requires reductant. Liquid chromatography-tandem mass spectrometry (LC-MS/MS) analysis reveals that MsrA-dependent Ubl conjugates are associated with DNA replication, protein remodeling, and oxidative stress and include the Ubl-modified MsrA, Orc3 (Orc1/Cdc6), and Cdc48d (Cdc48/p97 AAA+ ATPase). Overall, we found archaeal MsrA to have opposing MSO reductase and Ubl modifying activities that are associated with oxidative stress responses and controlled by exposure to mild oxidant.

## IMPORTANCE

Proteins that are damaged by oxidative stress are often targeted for proteolysis by the ubiquitin-proteasome system (UPS). The mechanisms that control this response are poorly understood, especially under conditions of mild oxidative stress when protein damage is modest. Here, we discovered a novel function of archaeal MsrA in guiding the Ubl modification of target proteins in the presence of mild oxidant. This newly reported activity of MsrA is distinct from its stereospecific reduction of methionine-*S*-sulfoxide to methionine residues. Our results are significant steps forward, first, in elucidating a protein factor that guides Ubl modification in archaea, and second, in providing an insight into oxidative stress responses that can trigger Ubl modification in a cell.

## INTRODUCTION

Cells incur oxidative stress as reactive oxygen species (ROS) are generated during metabolism or after exposure to physical/chemical factors such as ionizing radiation and desiccation (1). ROS can damage the proteins, lipids, nucleic acids, and carbohydrates of a cell. Particularly susceptible to oxidation by ROS are the metal centers and sulfur-containing amino acid residues (methionine [Met] and cysteine) of proteins (2). Proteins that are mildly oxidized can be repaired by chaperone-assisted refolding and/or by specialized repair enzymes as exemplified by methionine sulfoxide (MSO) reductases of the MsrA and MsrB (MsrA/B) type (3). MsrA/B reductases are widespread in all domains of life; MsrA catalyzes the reduction of free and protein-based methionine-*S*-sulfoxide to methionine, while MsrB reduces methionine-*R*-sulfoxide to methionine on proteins (4). Electrons for these reductions are provided by NAD(P)H through the activity of thioredoxin or by other factors (5). Proteins damaged beyond repair form aggregates or are degraded by redox-controlled proteolytic systems (6).

In eukaryotes, the ubiquitin (Ub) proteasome system (UPS) is a proteolytic nanocompartmentalized machine that is coordinated with redox signaling to ensure proteostasis. The UPS removes proteins damaged by oxidation and coordinates the timely turnover of proteins that serve as redox switches (6). Proteins targeted by the UPS are covalently linked to polymeric chains of ubiquitin (Ub) which can tag the protein for destruction by proteasomes (7). These Ub linkages are catalyzed by a process termed ubiquitinylation in which an E1 activates the Ub and E2 conjugating and E3 ligase enzymes guide the Ub to its target protein (8). The Ub-tagged proteins are recognized, unfolded, and destroyed by 26S proteasomes that use ATP to drive the process (9). Proteins unfolded by extreme oxidative insult can be destroyed by 20S proteasomes independently of ubiquitylation (10). The regulatory mechanisms used to sense and transmit the extent of protein damage by ROS to the Ub modification system are poorly understood.

*Archaea* have systems related to eukaryotic UPS. These archaeal UPSs are composed of a network of AAA ATPases, 20S proteasomes, and a ubiquitin-like (Ubl) modification system (11). The archaeal Ubl modification system relies upon an E1 enzyme to covalently attach small archaeal ubiquitin-like modifier proteins (SAMPs) to the lysine residues of target proteins by a process termed sampylation that operates in the absence of apparent E2/E3 homologs (12–14). Proteins covalently modified by the SAMPs are destroyed by proteasomes (15, 16) or stably inactivated (17).

The archaeon *Haloferax volcanii* has three SAMPs (SAMP1/2/3), which are covalently attached to target proteins, and a single E1 enzyme (UbaA) (12, 18, 19). UbaA can mediate autosampylation in its purified form (14) but is not known to directly modify target proteins, suggesting that additional factors are needed. Of the SAMPs, SAMP1 is associated with oxidative stress and is covalently attached to MsrA/B, the MSO reductase homologs of this archaeon (19). Here we report that MsrA switches from an MSO reductase to a protein factor that directs the sampylation of target proteins by the E1 UbaA in the presence of the mild oxidant dimethyl sulfoxide (DMSO). Our findings have implications regarding the convergent evolution of MsrA and the MsrB-like substrate binding domain of the eukaryotic DDB1-CRBN (Cereblon) E3 Ub ligase.

## RESULTS AND DISCUSSION

### MsrA is required for sampylation induced by DMSO.

**FIG 1:**
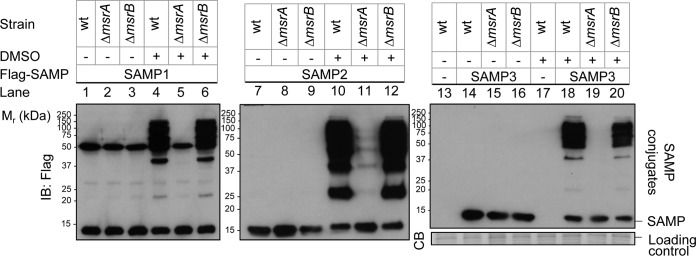
MsrA is important for DMSO-induced sampylation. *Hfx. volcanii* H26 parent (wt, wild type), YM1005 (Δ*msrA*), and YM1006 (Δ*msrB*) strains ectopically expressed Flag-SAMP1 (lanes 1 to 6), Flag-SAMP2 (lanes 7 to 12), and Flag-SAMP3 (lanes 14 to 16 and 18 to 20 or an empty vector control [-, lanes 13 and 17]) as indicated on top and top left. Strains were grown in ATCC 974 medium supplemented with 25 mM DMSO (+) or in mock control medium (−) as indicated on top left. Cell lysate was separated by reducing 12% SDS-PAGE and analyzed by anti-StrepII immunoblotting (IB) and Coomassie blue (CB) staining as indicated on bottom left. Migration of the molecular weight markers (*M*_r_) is indicated on the left. Migration of the SAMP and SAMP conjugates is indicated on the right. See Materials and Methods for details.

**FIG 2:**
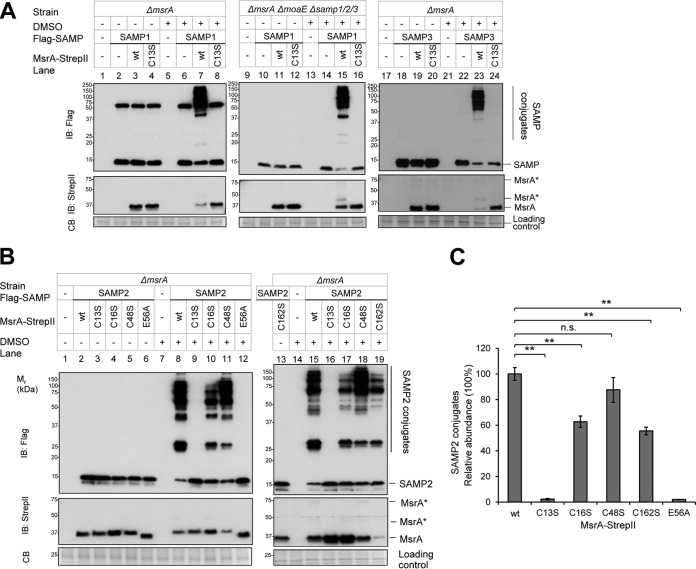
Conserved amino acid residues of MsrA that impact DMSO-induced sampylation. (A and B) *Hfx. volcanii* YM1005 (Δ*msrA*) and XF124 (*ΔmsrA ΔmoaE Δsamp1*/*2*/*3*) strains ectopically expressed Flag-SAMP1/2/3 and MsrA-StrepII (wt or unmodified, C13S, C16S, C48S, E56A, and C162A) as indicated on top left. Strains were grown in ATCC 974 medium with 25 mM DMSO (+) or in mock control medium (−) as indicated on top left. Lysate of stationary-phase cells (OD_600_, 2.0 to 3.0) was separated by the use of reducing 12% SDS-PAGE and analyzed by immunoblotting (IB). Molecular weight standards (*M*_r_) and the method of protein detection by anti-Flag and anti-StrepII IB and Coomassie blue staining (CB) are indicated on bottom left. Migration of SAMP2, SAMP2 conjugates, MsrA, and covalently modified MsrA (MsrA*) is indicated on right. (C) Histogram showing the relative abundances of SAMP2 conjugates migrating between 30 and 150 kDa from triplicate immunoblots as represented in panel B. Data represent means ± SD (*n* = 3) of results (**, *P* < 0.001; n.s., not significant). *P* values were determined by two-tailed, unpaired Student’s *t* test. The protein abundance is quantified by ImageJ. See Materials and Methods for details. wt, wild type.

MsrA/B are covalently linked to SAMP1 in *Hfx. volcanii* cells treated with the mild oxidant DMSO (19). To further understand this previous finding, *Hfx. volcanii ΔmsrA* and *ΔmsrB* mutants were generated through homologous recombination and analyzed for SAMP conjugates by immunoblotting (Fig. 1). To our surprise, the *ΔmsrA* mutant was found to be severely impaired in the level of SAMP1/2/3 conjugates that formed in the presence of DMSO, compared to the parent (wt) strain and the *ΔmsrB* mutant (Fig. 1, lane 5 versus lanes 4 and 6 [SAMP1], lane 11 versus lanes 10 and 12 [SAMP2], and lane 19 versus lanes 18 and 20 [SAMP3]). The major SAMP conjugate that formed in the absence of DMSO was SAMP1-MoaE (the large subunit of molybdopterin synthase) (19) and was formed by a mechanism that was independent of MsrA based on detection of this conjugate in an *ΔmsrA* mutant compared to an *ΔmoaE* mutant strain (Fig. 2A, lane 3 versus lane 11). Ectopic expression of *msrA* in the *ΔmsrA* mutant restored the level of DMSO-stimulated SAMP conjugates to that seen with the wild-type (wt) strain (Fig. 2A, lanes 7, 15, and 23; Fig. 2B, lanes 8 and 15), revealing that the difference in conjugate abundance was indeed attributed to *msrA*.

### MsrA active site residues are required for sampylation induced by DMSO.

Conserved residues of *Hfx. volcanii* MsrA (HvMsrA) were next examined for their role in sampylation. On the basis of analogy to characterized MSO reductases (20, 21), (i) HvMsrA C13 is the conserved active site residue that mediates nucleophilic attack of MSO, (ii) E56 is the invariant glutamate residue thought to bind the MSO oxygen atom, and (iii) C16, C48, and C162 are the cysteine residues that likely recycle the active site C13 after MSO reduction. Thus, the conserved residues were modified through site-directed mutagenesis, and the resulting HvMsrA variants were expressed in the *ΔmsrA* mutant and examined for activity associated with sampylation by *in vivo* complementation assay. DMSO-induced sampylation was found to be undetectable when the C13S and E56A variants of HvMsrA were expressed in the *ΔmsrA* mutant (Fig. 2A, lane 16 [SAMP1] and lane 24 [SAMP3]; Fig. 2B, lanes 9, 16, and 12 [SAMP2]). In contrast, DMSO-induced sampylation was detected, but at a reduced level, when the following recycling cysteine variants of HvMsrA were expressed in the *ΔmsrA* mutant: MsrA C48S (Fig. 2B, lanes 11 and 18; Fig. 2C, 88% total), MsrA C16S (Fig. 2B, lanes 10 and 17; Fig. 2C, 63% total), and MsrA C162S (Fig. 2B, lane 19; Fig. 2C, 56% total). Thus, the active site nucleophile (C13) and invariant glutamine (E56) appeared to be crucial to the DMSO-stimulated sampylation mediated by HvMsrA, while the recycling cysteines (at least when examined individually) appeared to be less important.

### MsrA-dependent sampylation is specific to conditions of mild oxidative stress.

MsrA-dependent sampylation was next tested under a variety of growth conditions. Treatment of cells with MSO was found to stimulate the levels of SAMP2/3 (not SAMP1) conjugates (see Fig. S1A in the supplemental material, lanes 5 to 6). On the basis of analysis of SAMP2 conjugate levels, stimulation of sampylation by MSO was found to require HvMsrA and its active site nucleophile (C13) (Fig. S1A, lanes 15 and 24, respectively). In contrast, the DMSO-related compounds, dimethyl sulfide (DMS) and dimethyl sulfone (DMSO_2_), did not stimulate sampylation (Fig. S1B, lanes 1 to 16). The levels of the SAMP2 conjugates were increased by treatment of cells with the proteasome inhibitor bortezomib (as previously reported [18]); however, MsrA was not required for this process (Fig. S1B, lanes 17 to 24). Hypochlorite was found to be a potent oxidant of *Hfx. volcanii* based on the observed oxidation of thiol groups in cells treated with NaOCl compared to DMSO and H_2_O_2_ (Fig. S2A). NaOCl stimulated the levels of SAMP2 (not SAMP1/3) conjugates; however, this stimulation did not require MsrA (Fig. S2B, lane 15). Thus, the SAMP conjugate levels were increased under a variety of conditions, including exposure to mild oxidant (DMSO and MSO), hypochlorite, and proteasome inhibitor (bortezomib). HvMsrA was required only for the mild oxidant-induced increase in SAMP conjugate levels.

10.1128/mBio.01169-17.1 FIG S1  Effect of chemical treatments (MSO, DMS, DMSO_2_, and bortezomib) on SAMP conjugate levels in *Hfx. volcanii*. Download FIG S1, PDF file, 0.2 MB. Copyright © 2017 Fu et al. 2017 Fu et al. This content is distributed under the terms of the Creative Commons Attribution 4.0 International license.

10.1128/mBio.01169-17.2 FIG S2  Exposure of *Hfx. volcanii* to NaOCl results in oxidation of thiol groups and stimulation of SAMP2 conjugate levels in the cell. Download FIG S2, PDF file, 0.2 MB. Copyright © 2017 Fu et al. 2017 Fu et al. This content is distributed under the terms of the Creative Commons Attribution 4.0 International license.

### HvMsrA/B are active MSO reductases.

**FIG 3:**
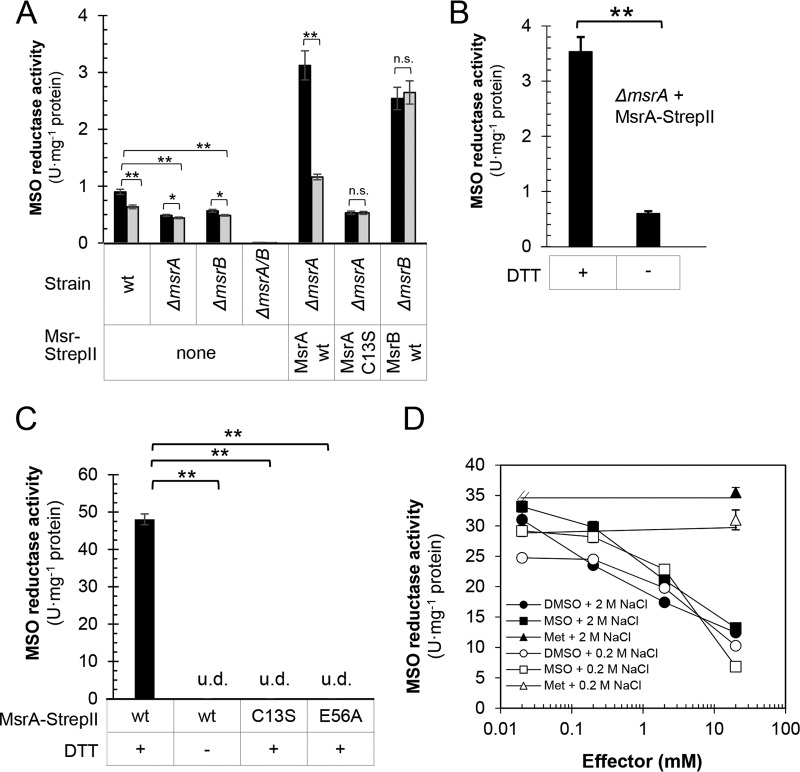
MSO-peptide reductase activity of MsrA is inhibited by DMSO. (A and B) MSO-peptide reductase activity of the cell lysate of *Hfx. volcanii* (H26 parent [wt], YM1005 [Δ*msrA*] and YM1006 [Δ*msrB*], and LR01 [*ΔmsrA ΔmsrB*] strains) carrying empty vector (none) or expressing MsrA-StrepII (wt and C13S) and MsrB-StrepII from plasmids, as indicated. (A) Cells were grown in ATCC 974 medium (black bars) and in ATCC 974 medium supplemented with DMSO (100 mM, gray bar). (B) MSO-peptide reductase activity was determined in the presence and absence of 20 mM DTT as indicated. (C and D) MSO-peptide reductase activity of purified MsrA (wt, C13S, and E56A) assayed in the presence and absence of 20 mM DTT as indicated. For panel D, the MsrA assay buffer was modified to include NaCl (0.2 to 2 M) and 0 to 20 mM effector (DMSO, MSO, and methionine [Met]) as indicated. U, units of activity (defined as nanomoles of dabsyl-MSO per minute). Data represent means ± SD of results (*n* = 3) (*, *P* < 0.05; **, *P* < 0.001; n.s., not significant; u.d., undetectable). *P* values were determined by two-tailed, unpaired Student’s *t* test. wt, wild type or parent (H26) strain. See Materials and Methods for details.

DMSO is a competitive inhibitor of the MSO-peptide reductase activity of yeast MsrA (22, 23), and yet this small molecule was required for HvMsrA to stimulate sampylation. To further understand this finding, the MSO-peptide reductase activity corresponding to MsrA was monitored in the lysate of cells grown in the presence and absence of DMSO. MSO-peptide reductase activity was found to be significantly reduced in the *ΔmsrA* mutant and to be restored to levels severalfold higher than those seen with the wt by ectopic expression of *msrA* versus the active site variant *msrA* C13S (Fig. 3A). Surprisingly, treatment of cells with DMSO was found to significantly reduce the levels of MSO-peptide reductase activity that corresponded to HvMsrA. This finding was particularly apparent in the *ΔmsrA* mutant that ecotopically expressed *msrA*, which had an over-2-fold reduction in MSO-peptide reductase activity under conditions of growth in the presence versus absence of DMSO (Fig. 3A). Omission of dithiothreitol (DTT) from the reaction mixture significantly impaired the MSO-peptide reductase activity attributed to HvMsrA (Fig. 3B). HvMsrB was also found to catalyze MSO-peptide reductase activity; however, the activity attributed to HvMsrB was not impaired by growth of cells in the presence of DMSO (Fig. 3A). Thus, HvMsrA/B are the major MSO-peptide reductases of *Hfx. volcanii*; however, the MSO-peptide reductase activity of HvMsrA is strikingly reduced in cells treated with DMSO.

To further understand these findings, HvMsrA was purified in its unmodified form (Fig. S3) and characterized for its MSO-peptide reductase activity. HvMsrA was found to catalyze the reduction of the MSO peptide (Fig. 3C), at levels comparable to those observed for MsrA of yeast (20, 24), mammals (25), and bacteria (26). The conserved active site residues of HvMsrA (C13 and E56) and DTT were found to be required for the MSO-peptide reductase activity (Fig. 3C). The mild oxidants DMSO and MSO inhibited the MSO-peptide reductase activity of HvMsrA in a dose-dependent manner, while methionine (Met) had no effect (Fig. 3D). Finding that DMSO inhibited the MSO-peptide reductase activity of HvMsrA contrasted with our *in vivo* results, which demonstrated HvMsrA to be important for DMSO-stimulated sampylation. We note that DMSO promotes the nonenzymatic oxidation of Met to MSO in the presence of a strong acid (1 to 6 M HCl) but does not do so in the absence of this acid (27). Thus, DMSO appeared to directly inhibit the MSO-peptide reductase activity of HvMsrA, potentially through a competitive inhibition mechanism similar to that seen with yeast MsrA, which can reduce DMSO to DMS (22, 23).

10.1128/mBio.01169-17.3 FIG S3  *Hfx. volcanii* MsrA proteins purified from recombinant *E. coli*. Download FIG S3, PDF file, 0.1 MB. Copyright © 2017 Fu et al. 2017 Fu et al. This content is distributed under the terms of the Creative Commons Attribution 4.0 International license.

### MsrA-dependent sampylation is reconstituted *in vitro*.

**FIG 4:**
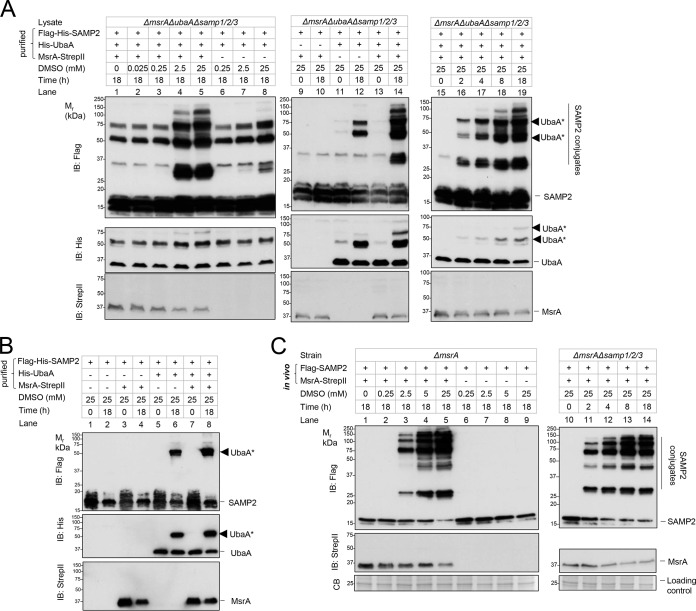
MsrA-dependent sampylation by *in vitro* reconstitution (A and B) compared to *in vivo* assay results (C). (A and B) Purified MsrA-StrepII was incubated with Flag-His-SAMP2, His-UbaA, and ATP (4 mM) for 0 to 18 h at 45°C. DMSO concentrations were adjusted from 0 to 25 mM. In the experiments represented by panel A, the reaction mixtures were supplemented with cell lysate of *Hfx. volcanii* LR03 (*ΔmsrA ΔubaA Δsamp1*/*2*/*3*, a sampylation-deficient strain). (C) In panel C, *Hfx. volcanii* YM1005 (Δ*msrA*) and LR02 (Δ*msrA* Δ*samp1*/*2*/*3*) strains expressed Flag-SAMP2 and MsrA-StrepII from plasmids. The cells were inoculated from log phase into ATCC 974 medium supplemented with 0 to 25 mM DMSO and grown for 0 to 18 h, as indicated. The samples represented in all panels were separated by reducing 12% SDS-PAGE and analyzed by anti-StrepII, anti-Flag, and/or anti-N-terminal His immunoblotting (IB) and Coomassie blue (CB) staining as indicated on the left. Data corresponding to migration of the molecular weight standards (*M*_r_) are indicated on the left. Data corresponding to migration of SAMP2, SAMP2 conjugates, MsrA, UbaA, and sampylated UbaA (see arrowhead [UbaA*]), the latter modified independently of MsrA, are noted on the right. See Materials and Methods for details.

Purified HvMsrA was next examined for its ability to reconstitute sampylation with the E1 UbaA and SAMPs by *in vitro* assay. Omitting HvMsrA or DMSO from the assay, the major SAMP conjugates detected were found to correspond to automodified forms of the E1 UbaA at 50 and 75 kDa (based on a previous study [14] and the results of the immunoblotting analysis performed in this study [Fig. 4A, lanes 1 and 12]). The monosampylated form of UbaA (at 50 kDa) was also detected when the reaction was simplified to include only UbaA, ATP, SAMP, and DMSO (Fig. 4B, lane 6). When HvMsrA was introduced into the original reconstitution assay, SAMP conjugates distinct from automodified UbaA (Fig. 4A, lane 12) were observed to form in the presence of DMSO (Fig. 4A, lanes 4 to 5) that were not detected in the absence of this mild oxidant (Fig. 4A, lane 1). This reconstitution of MsrA-dependent sampylation was found to closely correlate with the *in vivo* results (Fig. 4C), including the SDS-PAGE profile of the SAMP conjugates, the concentration of DMSO needed to trigger the response (2.5 mM), and the reaction time (8 h). We note that MsrA was purified from recombinant *Escherichia coli* by affinity and gel filtration chromatography to ensure a uniform preparation of enzyme that was devoid of any protein contaminants from *Hfx. volcanii*.

### Factors important for reconstitution of MsrA-dependent sampylation.

Other factors which could influence the reconstitution of MsrA-dependent sampylation were next examined. Genome-encoded levels of UbaA were used to minimize the detection of E1-mediated autosampylation by the immunoblotting assay. By this approach, the reductant (DTT) was not required (Fig. S4A, lanes 2 versus 4), while the conserved active site C13 was needed, for the HvMsrA-mediated sampylation reaction to proceed (Fig. S4A, lanes 6 and 10). ATP was also shown to be important (Fig. S4B, lanes 2 and 10), most likely due to the need for ATP to initiate sampylation through the E1 UbaA-mediated adenylation of the Ubl SAMP (14, 28). DMS and DMSO_2_ could not substitute for DMSO in stimulating the sampylation reaction (Fig. S4C). The products of the *in vitro* reconstitution reaction, while resistant to boiling with SDS and DTT, were sensitive to hydrolysis by HvJAMM1 (Fig. S4D, lanes 2 and 6), a JAMM/MPN+ metalloprotease that cleaves Ub/Ubl isopeptide linkages with precision (17, 29). These features provided additional support for the idea that the products of the *in vitro* reconstitution reaction were SAMP conjugates. Overall, MsrA, along with the E1 UbaA, was found to be an essential factor in reconstituting the SAMP conjugates that specifically formed in the presence of DMSO.

10.1128/mBio.01169-17.4 FIG S4  SAMP conjugates formed by the *in vitro* reconstitution assay are optimized (A to C) and cleaved by the JAMM/MPN+ metalloprotease HvJAMM1 (D). Download FIG S4, PDF file, 0.3 MB. Copyright © 2017 Fu et al. 2017 Fu et al. This content is distributed under the terms of the Creative Commons Attribution 4.0 International license.

### MsrA K176 and Orc3 K257 are sampylated *in vitro*.

**FIG 5:**
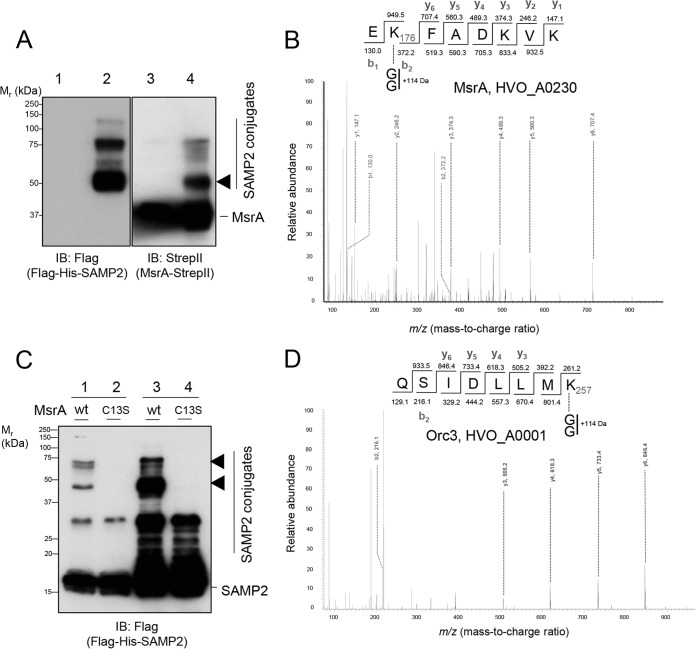
MsrA and Orc3 are conjugated to SAMP2 by the MsrA-dependent sampylation system. (A) Purified MsrA-StrepII was mixed with His-UbaA (E1), Flag-SAMP2 (Ubl), ATP, DMSO, and cell lysate of LR03 (*ΔmsrA ΔubaA Δsamp1*/*2*/*3*, an *Hfx. volcanii* strain deficient in sampylation). Mixtures were immediately quenched on ice (lanes 1 and 3) or incubated for 10 h at 45°C (lanes 2 and 4). MsrA-StrepII and its associated proteins were purified from the mixtures by the use of Strep-Tactin resin. Samples were separated by reducing 12% SDS-PAGE and analyzed by anti-Flag, and anti-StrepII immunoblotting (IB) as indicated. The SDS-PAGE gel slice of 50 kDa (arrowhead) was excised, and the proteins were digested with trypsin and analyzed by CID LC-MS/MS. Migration of the molecular weight standards (*M*_r_) is indicated on the left, while migration of SAMP2 and SAMP2 conjugates is indicated on the right. (B) Representative MS/MS spectra of the MsrA peptide found isopeptide linked to SAMP2. See panel A and the corresponding legend for details on the sample used for analysis. (C) Purified MsrA-StrepII (wt or C13S) and Flag-His-SAMP2 and cell lysate of LR02 (*ΔmsrA Δsamp1*/*2*/*3*, an *Hfx. volcanii* strain deficient in formation of SAMPs but retaining E1 UbaA function) were incubated with 4 mM ATP and 25 mM DMSO for 10 h at 45°C. Samples were directly separated by reducing 12% SDS-PAGE (lanes 1 to 2) or purified by complete His tag resin (lanes 3 to 4) prior to SDS-PAGE. Anti-Flag immunoblotting was used to guide excision of the protein bands (arrowhead) from SDS-PAGE gels for analysis by CID LC-MS/MS. (D) Representative MS/MS spectra of the Orc3 peptide found isopeptide linked to SAMP2. See panel C and the corresponding legend for details on the sample used for analysis. Sampylation sites (MsrA K176 and Orc3 K257) were based on detection of a +114-Da mass increase due to the tryptic remnant of the C-terminal diglycine of SAMP2. The y-ion and b-ion series detected are indicated (at a probability of >99.9% and an FDR of <0.1% for the peptide). See Materials and Methods for details.

HvMsrA is sampylated in *Hfx. volcanii* (19); thus, it was examined as a target of sampylation by the *in vitro* assay. Unmodified HvMsrA was incubated in the reconstitution assay and then purified from the reaction. Sampylated forms of MsrA were detected by immunoblotting assay, including a 50-kDa species that was abundant and 13 kDa larger than unmodified MsrA, suggesting that it was monosampylated (Fig. 5A). To further characterize this 50-kDa species, the protein was excised from the gel, digested with trypsin, and analyzed by collision-induced dissociation reversed-phase liquid chromatography–tandem mass spectrometry (CID LC-MS/MS). The protein was identified as MsrA (Fig. S5) and found to be isopeptide linked to SAMP2 at K176 (Fig. 5B).

10.1128/mBio.01169-17.5 FIG S5  Detection of MsrA peptides from the 50-kDa band after *in vitro* reconstitution assay by liquid chromatography-tandem mass spectrometry. Download FIG S5, PDF file, 0.1 MB. Copyright © 2017 Fu et al. 2017 Fu et al. This content is distributed under the terms of the Creative Commons Attribution 4.0 International license.

Other targets of the MsrA-dependent sampylation pathway were similarly analyzed after *in vitro* reconstitution. The sampylated proteins (generated *in vitro*) were enriched by affinity chromatography, excised from gels, digested with trypsin, and analyzed by CID LC-MS/MS (Fig. 5C and D). To control for MsrA-independent SAMP conjugates, reactions with the conserved active site variant of HvMsrA (C13S) were processed in parallel to HvMsrA (wt, unmodified). Orc3 (HVO_A0001) was found to be isopeptide linked to SAMP2 at K257 in reactions specific to HvMsrA (wt) (Fig. 5C and D). Orc3 is an Orc1/Cdc6-type DNA replication protein encoded on the pHV4 megaplasmid and is distinct from Orc1 encoded on the main chromosome (30). Finding Orc3 sampylated in an MsrA-dependent manner suggests that aspects of DNA replication may be altered under conditions of mild oxidative stress.

### Global analysis of proteins associated with the MsrA-dependent sampylation system.

To gain a global perspective on the proteins modified by the MsrA-dependent sampylation system, SAMP conjugates were purified from parent and *ΔmsrA* mutant strains grown in medium supplemented with DMSO (Fig. S6A) and analyzed by CID LC-MS/MS (Fig. S6B; Table 1). The AAA ATPase Cdc48d, while detected in both strains, was found to be linked to SAMP1 via an isopeptide at K350 only in the parent (Fig. S6B). Homologs of DNA replication, protein remodeling, and oxidative stress proteins were also found to be unique to the SAMP conjugates that were purified from the parent strain (versus the *ΔmsrA* mutant) (Table 1). The homologs included MsrA, SAMP1/3, and the redox-sensitive YhaK as well as proteins corresponding to Fe/S homeostasis (MoeA2, SufC, and ferritin-related protein), protein remodeling (thermosome Cct1/2, DnaK, Cdc48a/d, and peptidase S9), transcription (RpoB1), translation (AspS), DNA repair (Top6B), and metabolism (AldH2, PorB, AcaB4, and HutU) (Table 1). Of these, SAMP1/3, MsrA, and Cct2 are sampylated at target lysine residues as shown by previous studies (18, 19). Whether the proteins associated with the MsrA-dependent SAMP conjugates are poised for assisting in repair or targeted for degradation by the archaeal UPS remains to be determined. Finding that MsrA itself is sampylated by the system (Fig. 5A and B) and reduced in protein levels after exposure to DMSO (Fig. 2A, lanes 7, 15, and 23) supports the idea that at least a subset of these SAMP conjugates are targeted for destruction. The degradation of sampylated MsrA could serve to autoregulate the system. Extreme cellular damage by ROS may shift the balance of the SAMP conjugates (formed by the MsrA system during mild oxidative stress) to a state that can be degraded by proteasomes. Alternatively, the sampylated protein homologs associated with cellular homeostasis may be stably modified or inactivated by the MsrA system under conditions of mild oxidation. Stable modification could promote protein-protein interactions, similarly to SUMOylation (31), while stable inactivation could be reversed by precise HvJAMM1-mediated cleavage of the isopeptide bond linking the SAMP to its protein target (17). This reactivation may occur after severe oxidative insult when Fe-S homeostasis, protein remodeling, and DNA repair would be important.

10.1128/mBio.01169-17.6 FIG S6  SAMP1 conjugates purified from *Hfx. volcanii* parent (wt) and *ΔmsrA* mutant strains and analyzed by CID LC-MS/MS. Download FIG S6, PDF file, 0.5 MB. Copyright © 2017 Fu et al. 2017 Fu et al. This content is distributed under the terms of the Creative Commons Attribution 4.0 International license.

**TABLE 1:** Proteins identified by LC-MS/MS analysis to be associated with the SAMP1 conjugates purified from the parent strain compared to the *ΔmsrA* mutant strain^a^

Gene or protein	Uniprot ID	Protein description	Orthologous group	General function(s)	*M*_r_ (kDa)	Quantitative value (normalized total no. of spectra) for indicated strain		Coverage (%) for indicated strain		Sampylated site(s) (reference no.)
						*ΔmsrA*	Parent	*ΔmsrA*	Parent	
*aldH2*	D4GWU7	Aldehyde dehydrogenase	arCOG01252	[C] energy production and conversion	56	ND	1.3–3.2	ND	2.4–16	
*porB*	D4GXE9	Pyruvate-ferredoxin oxidoreductase, subunit beta	arCOG01599	[C] energy production and conversion	34	ND	5.7–12	ND	15–27	
	D4GUG3	Peptidase S9, prolyl oligopeptidase active site domain protein	arCOG01646	[E] amino acid transport and metabolism	67	ND	3.2–13	ND	4.1–19	
*moeA2*	D4GWD6	Molybdopterin molybdenum transferase	arCOG00217	[H] coenzyme transport and metabolism	66	ND	3.8–11	ND	5.7–16	
*acaB4*	D4GRI4	Acetyl-CoA C-acyltransferase	arCOG01278	[I] lipid transport and metabolism	39	ND	3.2–3.2	ND	13–20	
*aspS*	D4GT09	Aspartate—tRNA (Asp/Asn) ligase	arCOG00406	[J] translation, ribosomal structure and biogenesis	49	ND	1.3–3.9	ND	3.7–8.0	
*rpoB1*	D4GZX5	DNA-directed RNA polymerase subunit beta	arCOG01762	[K] transcription	68	ND	2.4–2.5	ND	4.9–8.0	
*top6B*	D4GZ00	Type 2 DNA topoisomerase 6 subunit B	arCOG01165	[L] replication, recombination and repair	87	ND	5.7–6.3	ND	5.3–6.0	
SAMP3	D4GVB0	Small archaeal modifier protein 3	ENOG4102TM3	[O] posttranslational modification, protein turnover, and chaperones	10	ND	4.7–14	ND	33–36	K18, K55, K62, K92 (18, 19)
SAMP1	D4GUF6	Small archaeal modifier protein 1	arCOG00536	[O] posttranslational modification, protein turnover, and chaperones; [H] coenzyme transport and metabolism	9	ND	23–138	ND	14–66	K4 (19)
*ctt1*	O30561	Thermosome subunit 1	arCOG01257	[O] posttranslational modification, protein turnover, and chaperones	59	ND–4.7	3.9–19	ND–4.5	10–14	
*ctt2*	O30560	Thermosome subunit 2	arCOG01257	[O] posttranslational modification, protein turnover, and chaperones	59	ND	15–20	ND	8.6–25	K280 (18)
*dnaK*	Q1XBW2	Chaperone protein DnaK	arCOG03060	[O] posttranslational modification, protein turnover, and chaperones	67	ND	5.7–11	ND	6.0–7.0	
*cdc48a*	D4GWM8	AAA-type ATPase (CDC48 subfamily)	arCOG01308	[O] posttranslational modification, protein turnover, and chaperones	82	ND	1.3–3.2	ND	5.9–10	K723 (S. Dantuluri et al., unpublished data)
*msrA*	D4GQQ9	Methionine-*S*-sulfoxide reductase	arCOG02816	[O] posttranslational modification, protein turnover, and chaperones	21	ND	32–64	ND	8.5–24	K108, K169, K172, K180, K182 (19)
*sufC*	D4GUK6	Fe-S cluster assembly ATPase SufC	arCOG04236	[O] posttranslational modification, protein turnover, and chaperones	33	ND	1.9–2.4	ND	9.0–14	
	D4GYM2	Ferritin related (IPR012347)	arCOG04687	[S] function unknown	21	ND	2.4–3.8	ND	15–17	
	D4GRD4	Redox-sensitive bicupin YhaK, Pirin superfamily	arCOG02935, COG1741	[S] function unknown	28	ND	3.2–6.3	ND	13–13	
*hutU*	D4GRM3	Probable urocanate hydratase	IPR023637	[S] function unknown	68	ND	2.4–3.2	ND	3.2–7.0	

a Proteins listed were found to be unique/enriched in the parent (H26, wt) strain compared to the *ΔmsrA* mutant strain at >99.0% probability with criteria of a 2-peptide minimum, FDR of <1% (protein), and FDR of <0.1% (peptide). CoA, coenzyme A; ND, not detected. The percent coverage and normalized total spectra are listed for two independent experiments. General-function data were assigned based on orthologous group assignment, with the exception of SAMP1/3 and SufC, which were manually assigned. The capital letter within the brackets is in reference to the aCOG group.

### Proposed model of MsrA-dependent Ubl modification.

Based on the results of this study, MsrA was found to be an integral component of the archaeal sampylation system under conditions of mild oxidative stress. We found MsrA and the UPS homologs to be important for survival of *Hfx. volcanii* in the presence of ROS, as *ΔmsrA*, *Δsamp2*, and *ΔubaA* mutants are hypersensitive to oxidants compared to parent and complemented strains (Fig. S7; Fig. S8). Based on the reconstitution of MsrA-dependent sampylation (in the presence of DMSO and irrespective of the presence of DTT), we propose that MsrA guides the Ubl SAMP to its protein target by a mechanism that is distinct from its MSO reductase activity (the latter of which is inhibited by DMSO and requires DTT). DMSO is suggested to induce a conformational change in MsrA that switches the enzyme from an MSO reductase to a component of the Ubl modification machinery. Interestingly, the thalidomide binding domain of cereblon is a structural homolog of MsrB that functions in ubiquitylation as a flexible substrate-presenting domain of the many diverse DCAFs (DDB1 and CUL4-associated factors) by functioning with E3 cullin 4-RING ligase CRL4 complexes (32, 33). MsrA and MsrB, while not sharing primary amino acid sequence homology, bind diverse protein substrates and have mirror-image active sites that are related through convergent evolution (34). Thus, we propose that MsrA serves as a protein substrate receptor that guides the formation of Ubl (SAMP) conjugates under conditions of mild oxidative stress in archaea.

10.1128/mBio.01169-17.7 FIG S7  Hypersensitivity of *Hfx. volcanii* methionine sulfoxide reductase mutants to oxidative stress. Download FIG S7, PDF file, 0.1 MB. Copyright © 2017 Fu et al. 2017 Fu et al. This content is distributed under the terms of the Creative Commons Attribution 4.0 International license.

10.1128/mBio.01169-17.8 FIG S8  Hypersensitivity of *Hfx. volcanii* ubiquitin-like modification system mutants (strains *ΔubaA* and *Δsamp2*) to oxidative stress. Download FIG S8, PDF file, 0.2 MB. Copyright © 2017 Fu et al. 2017 Fu et al. This content is distributed under the terms of the Creative Commons Attribution 4.0 International license.

## MATERIALS AND METHODS

### Strains and culture conditions.

Strains used in this study are listed in Table S1 in the supplemental material. *E. coli* TOP10 was used for routine selection, amplification, and maintenance of plasmid DNA. *E. coli* GM2163 was used for isolation of plasmid DNA prior to transformation of *Hfx. volcanii* as previously described (35). *E. coli* strains were grown in LB medium at 37°C. *Hfx. volcanii* strains were grown at 42°C in ATCC 974, glycerol minimal medium (GMM), and CA medium as previously described (36). Media was supplemented with ampicillin (Ap, 0.1 mg·ml^−1^), kanamycin (Km, 50 µg·ml^−1^), novobiocin (Nv, 0.2 μg·ml^−1^), and uracil (50 μg·ml^−1^) as needed. Solid medium was supplemented with 1.5 and 2.0% (wt/vol) agar for culture of *E. coli* and *Hfx. volcanii*, respectively. Liquid cultures were aerated by rotary shaking at 200 rpm. Cells were stored at −80°C in 20% (vol/vol) glycerol stocks. *Hfx. volcanii* strains were streaked from the freezer stocks onto ATCC 974 agar plates. Freshly isolated colonies of *Hfx. volcanii* were inoculated with a toothpick into 4 ml medium (13-by-100-mm culture tubes) and grown to log phase (optical density at 600 nm [OD_600_], 0.5 to 0.7) for use as an inoculum in the effector/stress test assays described below.

10.1128/mBio.01169-17.9 TABLE S1  List of strains and plasmids used in this study. Download TABLE S1, PDF file, 0.3 MB. Copyright © 2017 Fu et al. 2017 Fu et al. This content is distributed under the terms of the Creative Commons Attribution 4.0 International license.

### Effector/stress test assays.

To examine the influence of various compounds on sampylation, *Hfx. volcanii* cells that were grown to log phase in ATCC 974 medium (as above) were treated by 4 different approaches. (i) Cells were subcultured into ATCC 974 medium (4 ml) supplemented with 25 mM DMSO, DMS, or DMSO_2_ and grown to stationary phase (OD_600_, 2.0 to 3.0). (ii) Cells were washed with GMM (using centrifugation at 10,000 × *g* for 6 min at 25°C) and subcultured (0.1 ml) into GMM (4 ml). The GMM-grown cells were grown to log phase (OD_600_, 0.4 to 0.5), subcultured (0.1 ml) into GMM (4 ml) with 25 mM MSO, and then grown to stationary phase (OD_600_, 1.0 to 1.2). (iii) Cells were subcultured into ATCC 974 medium (4.4 ml), grown to log phase (OD_600_, 0.5 to 0.7), and treated with 0.1 mM bortezomib (2.2 µl of 200 mM bortezomib–99.8% [wt/vol] DMF [N,N-dimethylformamide]) for 24 h prior to harvest. (iv) Cells were subcultured into ATCC 974 medium (4 ml), grown to log phase (OD_600_, 0.5 to 0.7), and treated for 1 h with a final concentration of 8 mM NaOCl (Sigma-Aldrich, St. Louis, MO). All experiments included a mock control (the solvent that was used for delivery of the effector compound). Cells were harvested by centrifugation (10,000 × *g*, 6 min, 25°C) and analyzed by immunoblotting as described below. For stress test assays of the MSO reductase mutants, cells were inoculated from the freshly isolated colonies into GMM supplemented with 100 mM DMSO and grown to log phase. Cells were subcultured into the same medium (4 ml), harvested at stationary phase (OD_600_ of 1.5 to 2.0) by centrifugation (5,000 × *g*, 6 min, 25°C), and washed in 18% SW dilution buffer (per liter, 144 g NaCl, 18 g MgCl_2 ⋅ _6H_2_O, 21 g MgSO_4 ⋅ _7H_2_O, 4.2 g KCl, and 42 ml 1 M Tris-HCl; pH 7.5). Cells were adjusted to OD_600_ of 1.0, serially diluted in 18% SW dilution buffer, and spot plated (20 μl) onto GMM agar supplemented with DMSO, NaOCl, H_2_O_2_, and mock control medium as indicated. Plates were incubated at 42°C for 6 days. For survival assays of Ubl modification system mutants, ATCC 974-grown log-phase cells were subcultured into 4 ml of fresh ATCC 974 medium and grown to log phase (OD_600_, 0.4 to 0.6). Cultures were treated for 30 min with 16 mM NaOCl (Sigma-Aldrich) or a mock control. Cells were diluted to an OD_600_ of 0.04 and spot plated (20 μl) in 10-fold serial dilutions onto ATCC 974 agar medium. Plates were incubated at 42°C for 5 days.

### SDS-PAGE and immunoblotting analysis.

Cell pellets were resuspended in 1× reducing SDS loading buffer and boiled 3 times (for 5 min each time with 30 s of vortex mixing after the first and second times) prior to separation by SDS-PAGE; equivalent levels of protein loading for whole cells were determined on the basis of the OD_600_ of the cell culture (0.08 units per lane) and confirmed by Coomassie blue R-250 staining of parallel gels. Desalted protein samples were mixed with an equal volume of 2× SDS-PAGE loading buffer (100 mM Tris-Cl buffer at pH 6.8 with 4% [wt/vol] SDS, 20% [vol/vol] glycerol, 0.6 mg·ml^−1^ bromophenol blue, and 5% [vol/vol] β-mercaptoethanol). Samples were boiled for 10 min prior to separation by SDS-PAGE. Proteins were separated by reducing 12% SDS-PAGE (unless otherwise noted) and electroblotted onto polyvinylidene difluoride (PVDF) membranes (Amersham) per the standard protocol (BioRad). StrepII-tagged proteins were detected by the use of mouse anti-StrepII polyclonal antibody (Qiagen) followed by goat anti-mouse IgG (whole-molecule)-alkaline phosphatase-linked antibody (Sigma-Aldrich). Flag-tagged proteins were detected by the use of alkaline phosphatase-linked anti-Flag M2 monoclonal antibody (Sigma-Aldrich). His tagged proteins were detected using a monoclonal antibody unconjugated anti-His IgG2 antibody from mouse (27-4710-01; GE Healthcare) and alkaline phosphatase-linked goat anti-mouse IgG antibody (A5153; Sigma-Aldrich). Immunoreactive antigens were detected by chemiluminescence using CDP-Star (Applied Biosystems) as the alkaline phosphatase substrate and X-ray film (Hyperfilm; Amersham Biosciences, Inc.). The intensity of protein bands was quantified by ImageJ (37).

### DNA cloning and site-directed mutagenesis.

Plasmids and primers used in this study are listed in Table S1 and Table S2. Genes (*msrA* [HVO_A0230 or HVO_RS02870; UniProt gene identifier {ID}: 8923376] and *msrB* [HVO_2234 or HVO_RS15440; UniProt gene ID: 8925127]) were isolated by PCR using *Hfx. volcanii* parent (H26) genomic DNA as the template. Phusion DNA polymerase was used for high-fidelity PCR. *Taq* DNA polymerase was used for screening colonies by PCR. Site-directed mutagenesis was performed using a QuikChange II XL site-directed mutagenesis kit according to the supplier (Agilent Technologies). DNA sequencing of the expression plasmids was performed by Sanger automated DNA sequencing using an Applied Biosystems model 3130 genetic analyzer (ICBR Genomics Division, University of Florida).

10.1128/mBio.01169-17.10 TABLE S2  List of primers used in this study. Download TABLE S2, PDF file, 0.2 MB. Copyright © 2017 Fu et al. 2017 Fu et al. This content is distributed under the terms of the Creative Commons Attribution 4.0 International license.

### Generation of mutant, tag-integrant, and complement strains.

Target genes were deleted from the *Hfx. volcanii* chromosome by *pyrE2*-based homologous recombination (38, 39). Mutant strains were identified by PCR with primers outside the deletion plasmid sequence. Fidelity of the PCR products was confirmed by Sanger DNA sequencing (UF ICBR DNA sequencing core). Complementation of the mutation was performed by ectopic expression of the target gene from a pJAM202c-based shuttle plasmid.

### DTNB assay for free sulfhydryl groups.

Biological triplicates of the parent (H26) and *ΔmsrA* mutant (YW1005) strains were cultured twice to log phase in 4 ml GMM prior to inoculation to achieve a final OD_600_ of 0.015 in 25 ml GMM. Cultures (25 ml) were grown to stationary phase (OD_600_ of 1.5). Treatments were added at 0.2 ml per culture to final concentrations of 2.5 mM (NaOCl and H_2_O_2_) and 100 mM (DMSO). H_2_O alone served as the mock control. Following incubation for 20 min, cells were pelleted by centrifugation (3,900 × *g*; 4°C for 30 min) and washed in 5 ml Tris–salt buffer–7.5 (2 M NaCl, 50 mM Tris-Cl, pH 7.5). Cells were centrifuged again and resuspended in 3-ml reaction buffer (0.1 M sodium phosphate buffer [pH 8.0], 1 mM EDTA) supplemented with 5 μg·ml^−1^ DNase I (Sigma-Aldrich). Lysis was performed on ice by sonication (Fisher Scientific Model 500) at 30% amplitude (3 s on and 5 s off for 10 cycles in total). Lysate was clarified by centrifugation (13,000 × *g*; 4°C for 15 min). A bicinchoninic acid (BCA) assay (Pierce) was used to quantify total protein. Samples were diluted to a standardized concentration of 2 mg·ml^−1^ protein prior to assay. Ellman’s reagent solution was prepared by dissolving 4 mg DTNB [5,5′-dithio-*bis*-(2-nitrobenzoic acid)] (Thermo Scientific) per 1 ml of reaction buffer. Reactions were performed in borosilicate glass tubes (Fisher Scientific) (13-mm inner diameter by 100-mm length) containing 0.05 ml Ellman’s reagent solution, 2 mg protein, and reaction buffer to achieve a final volume of 2.8 ml. Reaction contents were subjected to gentle vortex mixing and incubated at room temperature (RT) for 15 min. The l-cysteine hydrochloride monohydrate (Fisher BioReagents) standard was dissolved in reaction buffer containing 5 μg·ml^−1^ DNase I and incubated similarly to the samples. Reaction mixtures were transferred in 0.25-ml aliquots, in triplicate, to a polystyrene 96-well plate (Fisher Scientific). Absorbance at 412 nm was measured (BioTek Synergy HTX). Available sulfhydryl groups were determined using the standard curve in experimental duplicate.

### Purification of MsrA.

MsrA proteins were purified from sampylation-deficient strain *Hfx. volcanii* LR03 (*Δsamp1 Δsamp2 Δsamp3 ΔmsrA ΔubaA*) carrying plasmid pJAM3010 (MsrA) and from *E. coli* Rosetta (DE3) carrying plasmid pJAM3200 (MsrA), pJAM2273 (MsrA C13S), and pJAM2284 (MsrA E56A). All of the MsrA proteins were fused to a C-terminal StrepII tag (-StrepII) to facilitate purification. The *Hfx. volcanii* strain was grown at 42°C to stationary phase in 4× ATCC 974 medium (1 liter) with Nv. The *E. coli* strains were freshly transformed with the plasmids and grown at 37°C in LB medium (0.5 liter) with Km. At the log phase (OD_600_ of 0.6 to 0.8), the *E. coli* cultures were shifted to 25°C and IPTG (isopropyl-β-d-thiogalactopyranoside) (0.4 mM) was added for 12 h prior to harvest. All cell types were harvested by centrifugation and lysed by the use of a French press (20,000 lb/in^2^) in lysis buffer composed of Tris–salt buffer–7.4 (50 mM Tris-Cl [pH 7.4], 2 M NaCl) supplemented with 1 mg·ml^−1^ EDTA-free protease inhibitor cocktail (Roche). Cell lysate was clarified by centrifugation and filtration (0.45-μm-pore-size surfactant-free cellulose acetate [SFCA]; Nalgene). Sample was applied to a Strep-Tactin column (GE Healthcare) (1-ml bed volume) equilibrated with Tris–salt buffer–7.4 at a flow rate of 0.5 ml·min^−1^. Unbound proteins were removed by washing the column with 140 ml Tris–salt buffer–7.4 at a flow rate of 1.2 ml·min^−1^. Proteins were eluted in Tris–salt buffer–7.4 supplemented with 5 mM d-desthiobiotin. Sample was further purified by gel filtration chromatography using a Superdex 75 10/300-Gl column (GE Healthcare) in Tris–salt buffer–7.5 at a flow rate of 0.3 ml·min^−1^.

### Methionine sulfoxide reductase assay.

MSO-peptide reductase activity of MsrA and cell extract was assayed as previously described (20). In brief, reaction mixtures (100 μl) containing 1 to 5 μg pure enzyme or 400 μg cell extract, 200 μM dabsyl-MSO, 20 mM DTT, and 25 mM Tris-Cl (pH 7.5) were incubated for 30 min at 37°C. Following the incubation period, the samples were treated with an equal volume of acetonitrile and subjected to C_18_ reversed-phase chromatography using high-performance liquid chromatography (HPLC). The peak of dabsyl-Met was quantified, and the specific activity was calculated accordingly. Cell extract for assay was prepared (as described above) from *Hfx. volcanii* strains grown to stationary phase in ATCC 974 medium with and without 100 mM DMSO and stored at −80°C before enzyme activity measurement. One unit of activity is defined as 1 nmol dabsyl-Met generated per min at 37°C under standard conditions. Assay protein concentrations were measured by the use of Bradford reagent (Bio-Rad), with bovine serum albumin (BSA) used as the standard.

### *In vitro* reconstitution of MsrA-dependent Ubl protein modification.

Reaction mixtures (100 µl total) contained 80 µl cell lysate (20 to 30 mg·ml^−1^), 5 µM MsrA-StrepII, 10 µM Flag-His-SAMP2, and/or 5 µM His-UbaA in assay buffer (50 mM Tris-Cl [pH 7.5], 4 mM ATP, 25 mM DMSO, 2 mM Mg^2+^, 0.5 mM DTT, and 2 M NaCl). Cell lysate was derived from *Hfx. volcanii* strain LR02 (Δ*samp1* Δ*samp2* Δ*samp3* Δ*msrA*), strain XF124 (Δ*samp1* Δ*samp2* Δ*samp3* Δ*msrA* Δ*moaE*), or strain LR03 (Δ*samp1* Δ*samp2* Δ*samp3* Δ*msrA* Δ*ubaA*) carrying pJAM947 plasmid (Flag-SAMP1) or pJAM949 plasmid (Flag-SAMP2). The strains were grown to stationary phase (OD_600_ of 3.5 to 4.0) in ATCC 974 medium (0.5 liter) supplemented with Nv and 25 mM DMSO as indicated. Cells were harvested by centrifugation (3,500 × *g*, 10 min at 15°C) and suspended and lysed by the use of a French press (20,000 lb/in^2^) in 8 ml of Tris–salt buffer–7.5 supplemented with 10 mM MgCl_2_, 100 µM bortezomib (LC Laboratories), and 30 μg·ml^−1^ DNase I from bovine pancreas (Sigma-Aldrich). Cell debris was removed by centrifugation and filtration (0.2 μm SFCA; Nalgene). The resulting cell lysate was dialyzed 3 times against Tris–salt buffer–7.5 at 4°C. To study the effect of different chemical agents on the stimulation of SAMP conjugate formation, buffer was supplemented with 4 mM ATP and 25 mM DMSO or related compounds (DMS, DMSO_2_, and MSO) as indicated. Reaction mixtures were incubated at 45°C for 0 to 18 h. To cleave the SAMP conjugates formed *in vitro*, HvJAMM1 (5 μM) (29) was added and the mixture was incubated for 4 h at 45°C; EDTA (50 mM) was included to inactivate the metalloprotease as a negative control. After assay, salts were removed from the reaction mixtures using Zeba Spin Desalting columns (7 K molecular weight cutoff [MWCO]) according to the instructions of the supplier (Thermo Scientific), and the reaction products were analyzed by reducing 12% SDS-PAGE and immunoblotting.

### Purification of proteins from the *in vitro* reconstitution assay.

MsrA-StrepII was purified from the *in vitro* reconstitution assay by use of Strep-Tactin Superflow resin (Qiagen). Aliquots (0.5 ml) of the assay were mixed with 2.5 ml Tris–salt buffer–7.4. The resulting sample (3 ml) was clarified by filtration (0.45 μm pore size, as described above) prior to application to 0.15 ml resin equilibrated in Tris–salt buffer–7.4. To enhance the yield of MsrA, the flowthrough of the sample was applied to resin one more time. Nonspecific proteins were removed by washing the resin with 40 column volumes of lysis buffer. Bound proteins were eluted by addition of 30 μl of 5 mM d-desthiobiotin dissolved in Tris–salt buffer–7.4. Sampylated proteins were purified from the *in vitro* reconstitution assay by use of Flag-His-SAMP2 as the substrate and complete His tag (Roche) as the purification resin. Resin was equilibrated in phosphate-buffered saline (PBS) (50 mM NaH_2_PO_4_, 300 mM NaCl, pH 8.0). Each sample (1.52 ml) was mixed with 8.48 ml PBS (with 5 mM EDTA, 5 mM DTT, 1 mg·ml^−1^ EDTA-free protease inhibitor cocktail [Roche], 50 mM NaH_2_PO_4_, 300 mM NaCl, pH 8.0) after the *in vitro* assay and applied to the His tag resin (100 µl). Nonspecific proteins were removed by washing the His tag resin with 40 column volumes of PBS. Bound proteins were eluted from the resin by addition of 80 μl of PBS supplemented with 200 mM imidazole. Elution fractions were mixed with equal volumes of 2× SDS reducing buffer and separated by SDS-PAGE. Proteins were analyzed in parallel gels by immunoblotting and staining for total protein by the use of SYPRO Ruby (Bio-Rad) followed by Bio-Safe Coomassie (Bio-Rad) as directed by manufacturer. The Ubl (SAMP2)-modified band of MsrA at 50 kDa was excised in gel slices. Similarly, the sampylated proteins at 50 and 75 kDa that were specific to the MsrA versus MsrA C13S reactions were excised.

### Purification of SAMP1 S85R conjugates.

The protocol used was as described by Dantuluri et al. (19) with the following modifications. The strains included the *Hfx. volcanii* parent (H26) and the *ΔmsrA* mutant (YM1005) expressing Flag-SAMP1 S85R on plasmid pJAM556. Log-phase cells were inoculated at an OD of 0.01 in 0.75 liters of ATCC 974 medium supplemented with 100 mM DMSO and cultured for 85 h. Cells were harvested by centrifugation (3,500 × *g*, 4°C for 15 min) and washed in 15 ml Tris–salt buffer–7.5. Cell pellets were stored at −80°C until further use. Biological replicates (2× 0.75-liter cultures [each strain]) were pooled and resuspended (10 ml per g wet weight) in lysis buffer comprised of Tris–salt buffer–7.4 supplemented with 1% Triton X-100, 1 mM EDTA, 5 μg·ml^−1^ DNase I, and protease inhibitor cocktail as directed by the supplier (Sigma-Aldrich). Lysis was carried out by three passages through a French pressure cell at 20,000 lb/in^2^. Cell lysate was clarified by centrifugation (13,000 × *g*, 4°C for 25 min) and filtration (0.45 μm pore size) as described above. Protein of cell lysate was quantified by BCA assay (Pierce) and applied (25 mg) to an anti-Flag column preequilibrated with TBS (150 mM NaCl, 50 mM Tris-Cl, pH 7.4). The column was 1 cm in diameter and contained 0.3 ml anti-Flag M2 affinity beads (Sigma-Aldrich). Bound proteins were washed in 20 column volumes of TBS prior to elution in 0.5 ml 3× Flag peptide (Sigma-Aldrich) (0.1 mg·ml^−1^)–TBS. Purified proteins were separated by reducing SDS-PAGE and stained with SYPRO Ruby (Bio-Rad) followed by Bio-Safe Coomassie (Bio-Rad) as directed by the manufacturer. Gel regions of approximately 30 to 45 kDa and 60 to 200 kDa were excised and analyzed by LC-MS/MS.

### LC-MS/MS analysis.

Gel pieces with protein bands of interest were washed twice with MilliQ water and destained with 50% (vol/vol) acetonitrile–50 mM ammonium bicarbonate buffer. After destaining, the gel pieces were dried using a CentriVap console (Labconco, USA) and reduced with 45 mM DTT at 55°C for 45 min. To prevent alkylation of lysine residues, the reduced protein samples were alkylated with 100 mM 2-chloroacetamide in the dark for 45 min at room temperature. The protein samples were washed 3 times with 50% (vol/vol) acetonitrile–50 mM ammonium bicarbonate buffer and digested with trypsin (Promega) (12.5 µg·µl^−1^) for 16 h at 37°C. Peptides were extracted from the gel pieces with 80% (vol/vol) acetonitrile containing 0.01% (vol/vol) trifluoroacetic acid. Peptides were isolated by the use of ZipTip pipette tips (Merck Millipore Ltd., Carrigtwohill, CO) according to the manufacturer’s instructions prior to MS.

The enzymatically digested samples were injected onto a capillary trap (LC Packing C_18_ Pep Map nanoflow HPLC column) (EASY-nLC 1000 Proxeon; Thermo Scientific) and desalted for 5 min with 0.1% (vol/vol) acetic acid (flow rate, 300 nl·ml^−1^). Peptide fragments were eluted by the use of a linear gradient for 30 min at 300 nl·ml^−1^ starting at 3% solvent A and 97% solvent B and finishing at 60% solvent A and 40% solvent B. Solvent A consisted of 0.1% (vol/vol) acetic acid, 3% (vol/vol) acetonitrile, and 96.9% (vol/vol) H_2_O. Solvent B consisted of 0.1% (vol/vol) acetic acid, 96.9% (vol/vol) acetonitrile, and 3% (vol/vol) H_2_O. MS/MS analyses of fractions were carried out on a Q Exactive Plus hybrid quadrupole-Orbitrap mass spectrometer (Thermo, Fisher Scientific). For the Q Exactive Plus procedure, a top 10 method was used. The ion spray voltage was set to 2,180 V. Full MS scans were acquired with a resolution of 70,000 from *m*/*z* 400 to 2,000. MS/MS scans were acquired with a resolution of *m*/*z* 17,500. The 20 most intense ions were fragmented by high-energy collisional dissociation (HCD). Dynamic exclusion was set to 60 s.

All MS/MS samples were analyzed using Mascot (Matrix Science, Inc., London, United Kingdom; version 2.4.1). Mascot was set up to search the database containing *Hfx. volcanii* DS2 proteins and MsrA-StrepII (8,089 entries), assuming the digestion with trypsin. The false-discovery rate (FDR) was specified at ≤1.0% using the automatic decoy database search in Mascot. Mascot was searched with a fragment ion mass tolerance of 0.1 Da and a parent ion tolerance of 10.0 ppm. Carbamidomethyl of cysteine was specified as a fixed modification. Gln to pyro-Glu of the N terminus, deamidation of Asn and Gln, oxidation of Met, and -Gly-Gly signatures were specified as variable modifications. Scaffold (version 4; Proteome Software Inc., Portland, OR, USA) was used to validate MS/MS-based peptide and protein identifications. Peptide identifications were accepted if established at >59.0% probability as specified by the Peptide Prophet algorithm (40). Protein identifications were accepted if established at >99.0% probability and corresponding to least two identified unique peptides, as assigned by the Protein Prophet algorithm (41).
