# Supplementary material for: Methionine Sulfoxide Reductase A (MsrA) and Its Function in Ubiquitin-Like Protein Modification in Archaea
Source: mBio. 2017 Sep 5;8(5):e01169-17. doi: 10.1128/mBio.01169-17 (PMC5587910; doi:10.1128/mBio.01169-17)
Supplement: FIG S1 [file mbo004173464sf1.pdf]

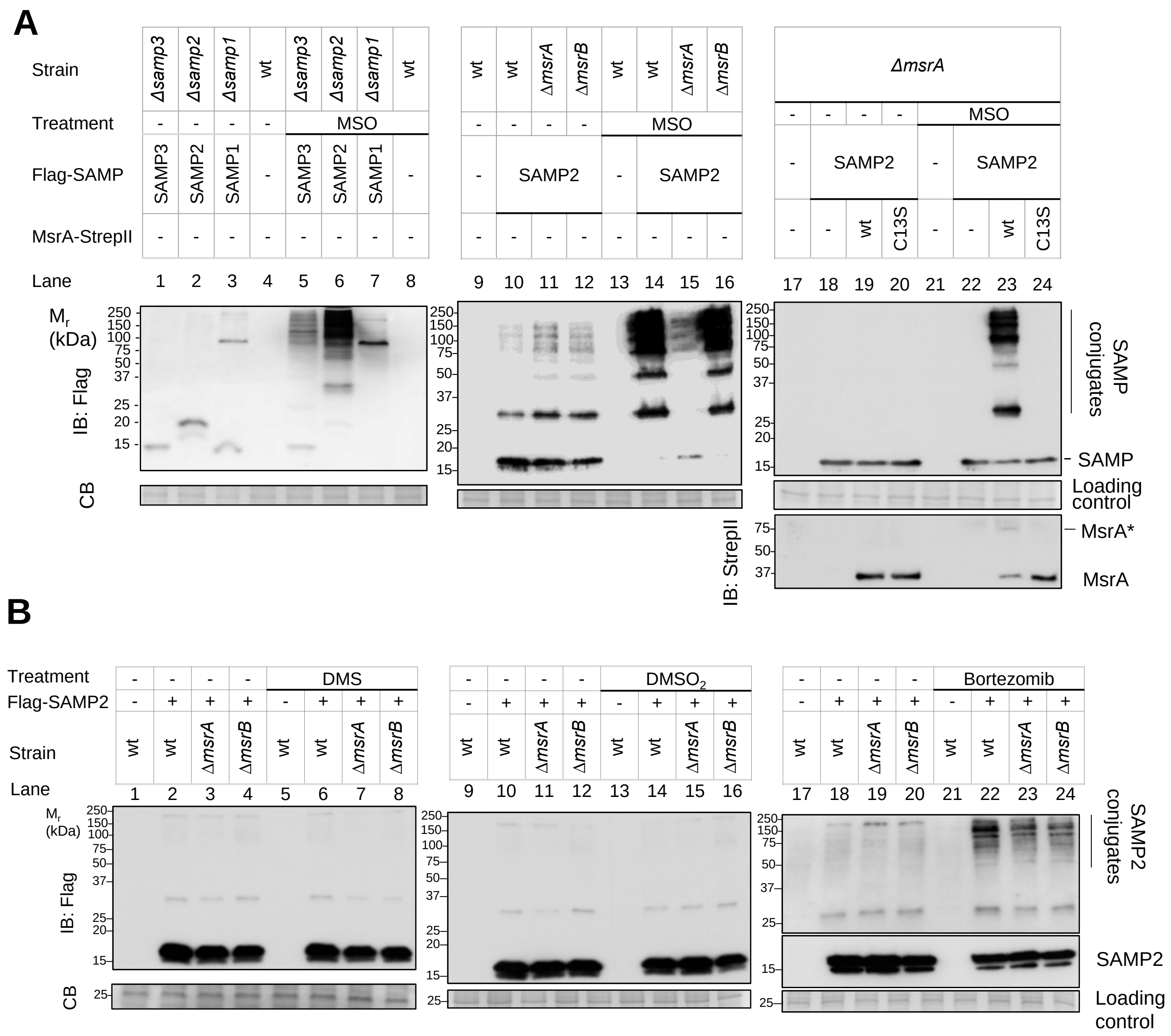

Supplemental Fig. S1. Effect of chemical treatments (MSO, DMS, $DMSO_2$ and bortezomib) on SAMP conjugate levels in *Hfx. volcanii*. *Hfx. volcanii* strains (parent H26, *ΔmsrA* YW1005, and *ΔmsrB* YW1006) expressing Flag-SAMP1/2/3 and MsrA-StrepII (wt and C13S) from plasmids were treated with 25 mM MSO (panel A, lanes 5-8, 13-16 and 21-24), 25 mM DMS (panel B, lanes 5-8), 25 mM $DMSO_2$ (panel B, lanes 13-16), and 0.1 mM bortezomib (panel B, lanes 21-24), as indicated. Cell lysate was separated by reducing 12% SDS-PAGE with molecular mass standards (M_r) indicated on left. Cell proteins were detected by anti-Flag immunoblotting (IB) analysis and Coomassie blue staining (CB) as indicated on left. Migration of SAMP2, SAMP2 conjugates, MsrA, and covalently modified MsrA* is indicated on the right. wt, wild type or parent. See methods for details.
