# Supplementary figures and images for: Methionine Sulfoxide Reductase A (MsrA) and Its Function in Ubiquitin-Like Protein Modification in Archaea
Source: mBio. 2017 Sep 5;8(5):e01169-17. doi: 10.1128/mBio.01169-17 (PMC5587910; doi:10.1128/mBio.01169-17)

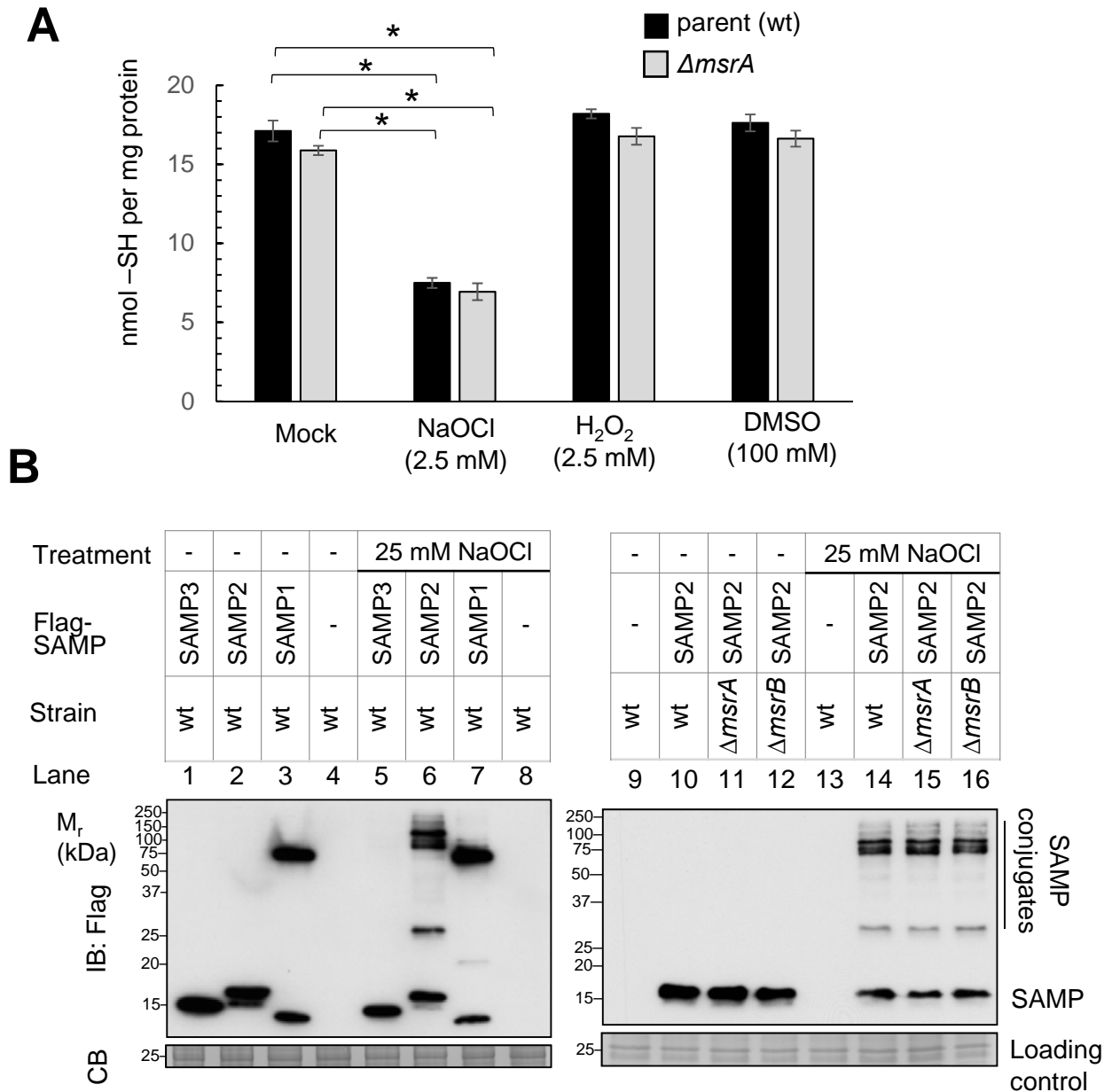

Supplement: FIG S2 [file mbo004173464sf2.pdf]
