# Supplementary material for: Methionine Sulfoxide Reductase A (MsrA) and Its Function in Ubiquitin-Like Protein Modification in Archaea
Source: mBio. 2017 Sep 5;8(5):e01169-17. doi: 10.1128/mBio.01169-17 (PMC5587910; doi:10.1128/mBio.01169-17)
Supplement: FIG S3 [file mbo004173464sf3.pdf]

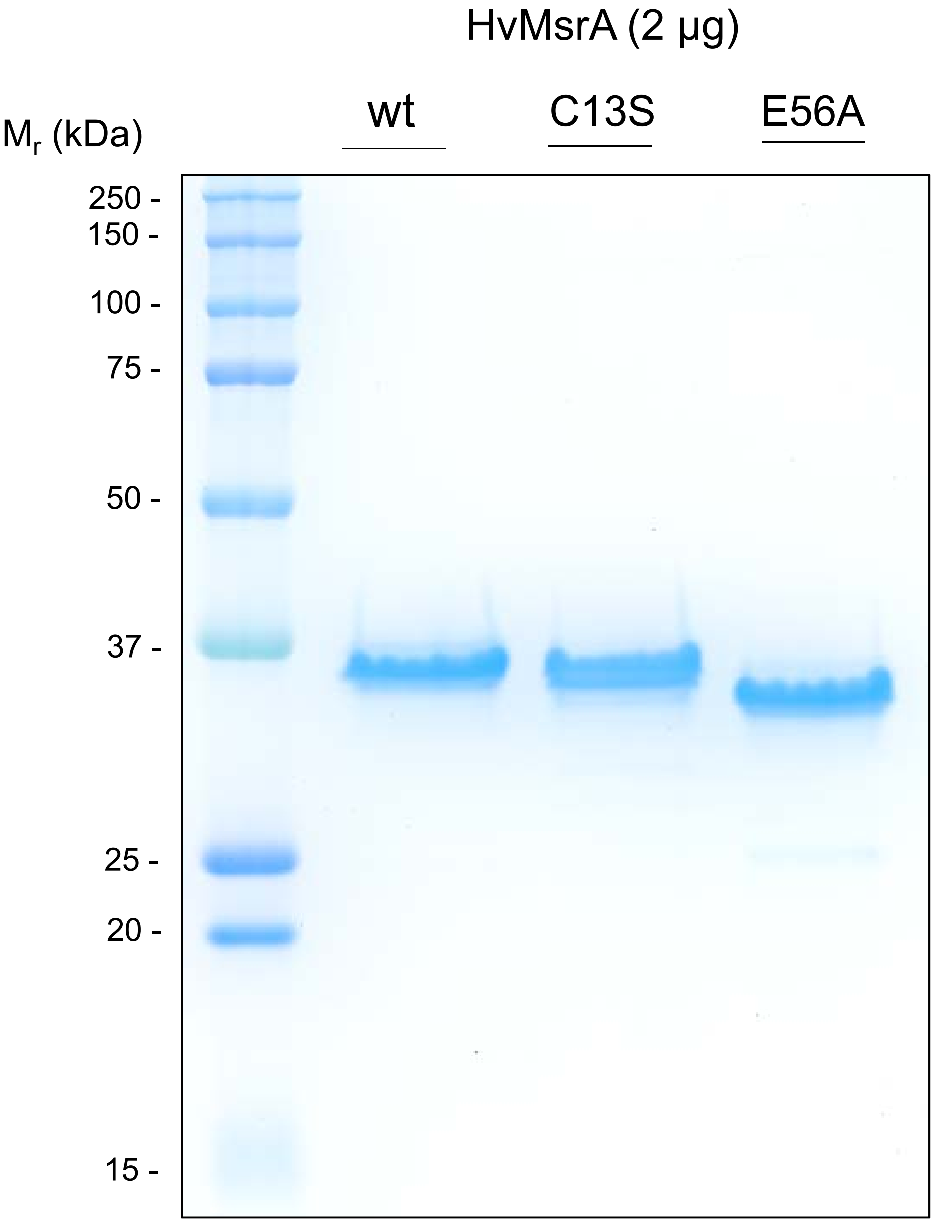

Supplemental Fig. S3. *Hfx. volcanii* MsrA proteins purified from recombinant *E. coli*. HvMsrA (wt, C13S and E56A) proteins were fused to a C-terminal StrepII tag and expressed in recombinant *E. coli.* Proteins were purified by StrepII affinity chromatography and gel filtration chromatography. Proteins were separated (2 µg per lane) by reducing 12 % SDS-PAGE and stained with Coomassie blue (CB). Molecular mass standards (M_r, kDa) are indicated on the left. wt, wild type protein.
