# Supplementary material for: Methionine Sulfoxide Reductase A (MsrA) and Its Function in Ubiquitin-Like Protein Modification in Archaea
Source: mBio. 2017 Sep 5;8(5):e01169-17. doi: 10.1128/mBio.01169-17 (PMC5587910; doi:10.1128/mBio.01169-17)
Supplement: FIG S4 [file mbo004173464sf4.pdf]

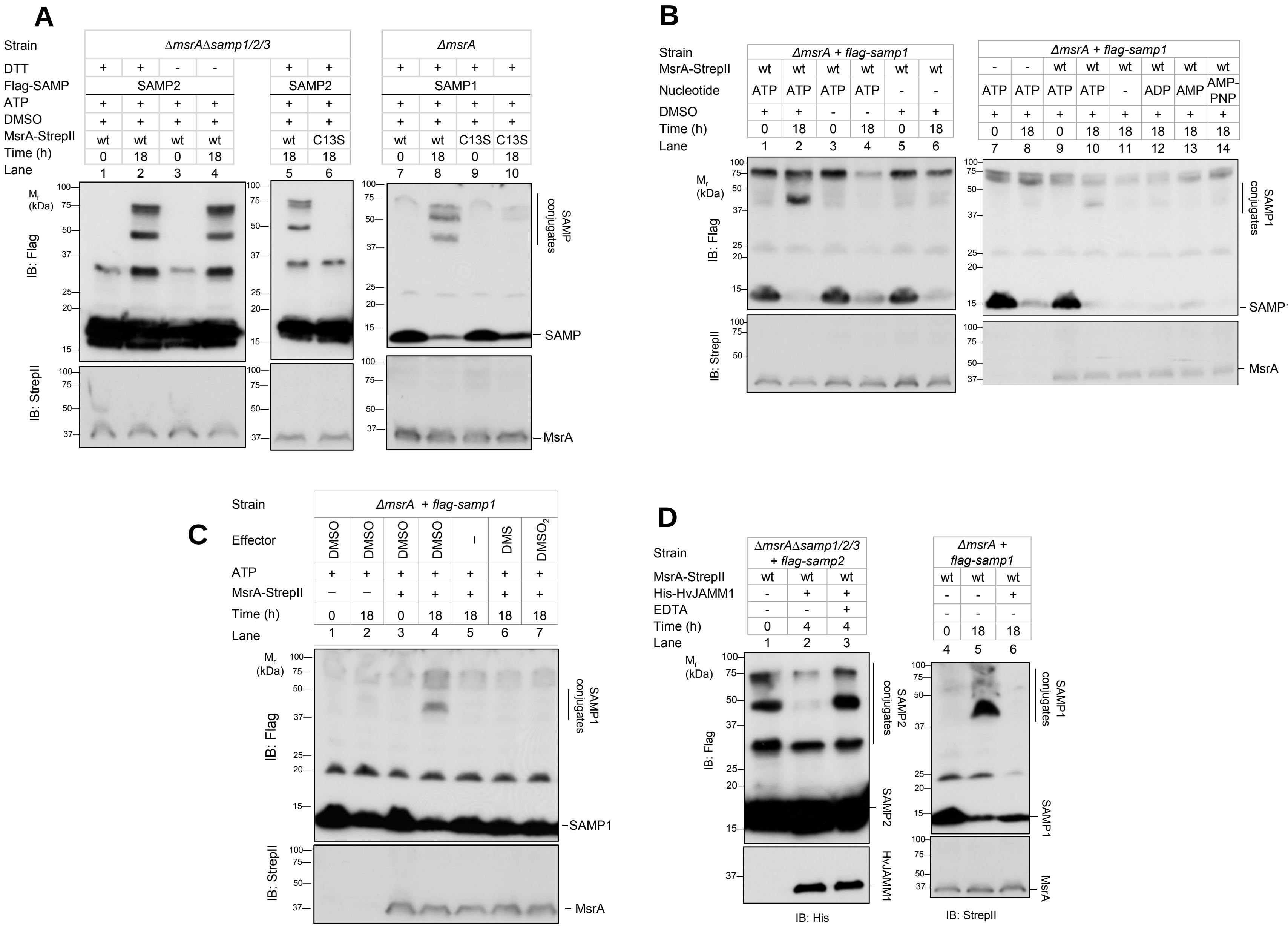

Supplemental Fig. S4. SAMP conjugates formed by the *in vitro* reconstitution assay are optimized (A-C) and cleaved by the JAMM/MPN+ metalloprotease HvJAMM1 (D). (A-C) Purified MsrA-StrepII (wt or C13S) was incubated in assay buffer with DTT (0.5 mM), effector (25 mM DMSO, DMS or $DMSO_2$), nucleotide (4 mM ATP, ADP, AMP or AMP-PNP) and cell lysate from strain LR02 (*ΔmsrAΔsamp1/2/3*) spiked with purified Flag-SAMP2 or strain YW1005 (*ΔmsrA*) expressing Flag-SAMP1 from plasmid pJAM947 for 0-18 h at 45°C, as indicated. D) SAMP conjugates formed by the *in vitro* assay were mixed with 5 μM HvJAMM1 (a JAMM/MPN+ metalloprotease that cleaves Ubl/Ub isopeptide bonds) in the presence and absence of EDTA (50 mM) as a control (to remove the active site Zn^{2+} from HvJAMM1). Proteins were separated by reducing 12% SDS-PAGE and analyzed by anti-StrepII, anti-N-terminal-His, anti-Flag immunoblotting (IB) and Coomassie blue staining (CB) as indicated on the left and bottoms of the images. Migration of molecular mass standards (Mr, kDa) is indicated on left. Migration of SAMPs, SAMP conjugates, MsrA and HvJAMM1 is indicated on right. wt, wild type. See methods for details.
