# Supplementary material for: Methionine Sulfoxide Reductase A (MsrA) and Its Function in Ubiquitin-Like Protein Modification in Archaea
Source: mBio. 2017 Sep 5;8(5):e01169-17. doi: 10.1128/mBio.01169-17 (PMC5587910; doi:10.1128/mBio.01169-17)
Supplement: FIG S5 [file mbo004173464sf5.pdf]

A

gi|201605191 (100%), 22,173.1 Da
MsrA with GT linker and StrepII tag
6 exclusive unique peptides, 11 exclusive unique spectra, 11 total spectra, 67/199 amino acids (34% coverage)

Theoretical coverage by trypsin digestion, 86/199 amino acids (43%)
Actual coverage (34%)

```
MGSTQTATFG GGCFWCVEAA FKELDGISEV TSGYAGGETE NPSYEQVCSG STGHAEVVQV
DYDPAVVGYD ELLDVFFAVH DPTQLNRQGP DVGTQYRSIV LFHDDEQKRQ ASAYIDALDE
EYDDEVVTEL VPLETFYEAE EHHQDYFEKV STADGNTNDA YCQFNVVPKI EKVREKFADK
VKAEAEPDAG TWSHPQFEK
```

B

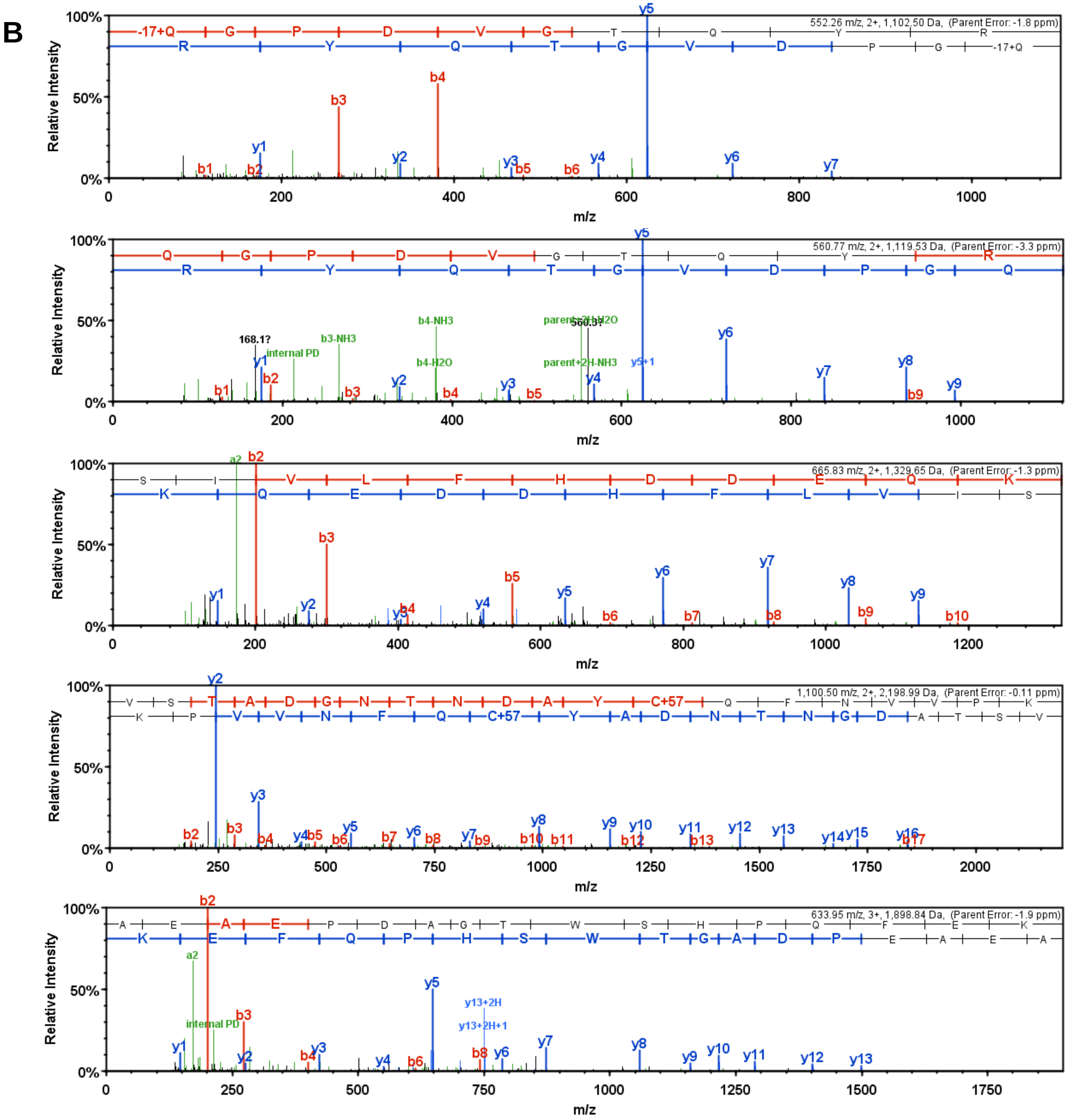

Supplemental Fig. S5. Detection of MsrA peptides from the 50 kDa band after *in vitro* reconstitution assay by liquid chromatography tandem mass spectrometry. A) Total 6 exclusive unique peptides of MsrA with 34% coverage were detected by LC-MS/MS analysis. The theoretical coverage after trypsin digestion is 43%. The peptides detected by LC-MS/MS were labeled by yellow. B) 5 representative MS/MS spectra of MsrA peptides derived by collision-induced dissociation of precursor after trypsin digestion. High mass accuracy MS/MS unambiguously confirmed MsrA identity based on the near complete matches of b- (colored red) and y-type ions (colored blue) derived from MsrA peptides. All the 5 representative MsrA peptides were detected with 100 % peptide identification probability. The MsrA peptide modified by SAMP2 is listed in Figure 5A. See methods for details.
