# Supplementary material for: Methionine Sulfoxide Reductase A (MsrA) and Its Function in Ubiquitin-Like Protein Modification in Archaea
Source: mBio. 2017 Sep 5;8(5):e01169-17. doi: 10.1128/mBio.01169-17 (PMC5587910; doi:10.1128/mBio.01169-17)
Supplement: FIG S6 [file mbo004173464sf6.pdf]

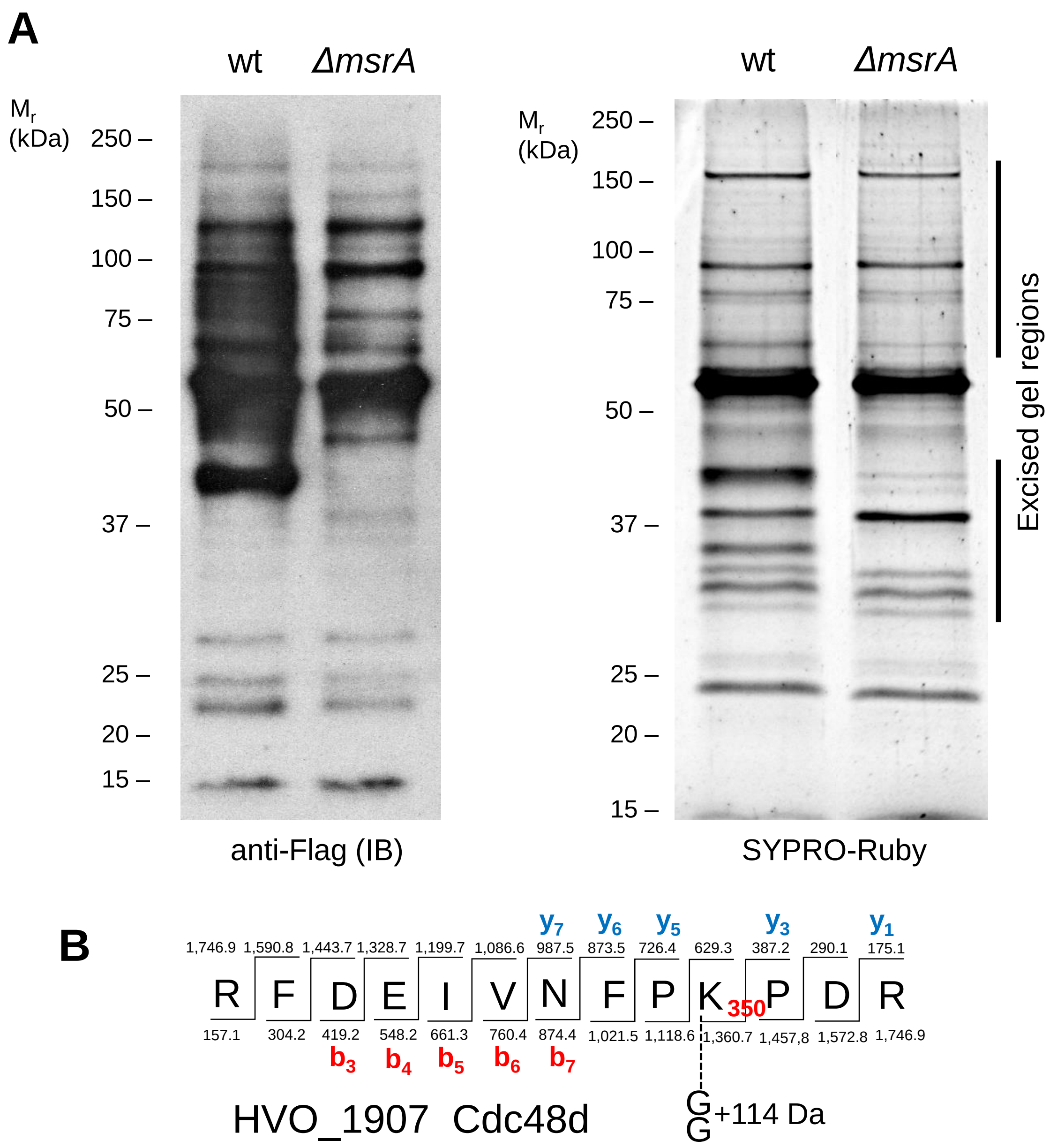

Supplemental Figure S6. SAMP1 conjugates purified from *Hfx. volcanii* parent (wt) and *ΔmsrA* mutant strains and analyzed by CID LC-MS/MS. (A) SAMP conjugates were purified by anti-Flag affinity chromatography from *Hfx. volcanii* parent (wt, H26) and *ΔmsrA* (YW1005) mutant strains, as indicated, with both strains expressing Flag-SAMP1 S85R from plasmid pJAM556. Proteins were separated by reducing 10% SDS-PAGE and analyzed by anti-Flag immunoblotting (IB, left panel) and SYPRO-Ruby protein stain (BioRad, right panel). Migration of the molecular mass standards (M_r kDa) is indicated on the left. Vertical bars positioned right indicate the portions of the gel that were excised from the parent and *ΔmsrA* lanes and analyzed by LC-MS/MS. (B) Schematic of the y- and b-ion series detected by CID LC-MS/MS of a tryptic peptide of Cdc48d modified by SAMP1 S85R. Sample was derived from panel A. The Ubl modification, which was found unique to the parent (vs. *ΔmsrA*), was detected based on a +114 Da mass increase due to attachment of a tryptic remnant of the C-terminal diglycine of SAMP1 S85R. The calculated masses are shown above each y- and b-ion with those in blue and red, respectively, detected at high probability (> 99.9%) and low FDR (< 0.1%).
