# Supplementary material for: Methionine Sulfoxide Reductase A (MsrA) and Its Function in Ubiquitin-Like Protein Modification in Archaea
Source: mBio. 2017 Sep 5;8(5):e01169-17. doi: 10.1128/mBio.01169-17 (PMC5587910; doi:10.1128/mBio.01169-17)
Supplement: FIG S7 [file mbo004173464sf7.pdf]

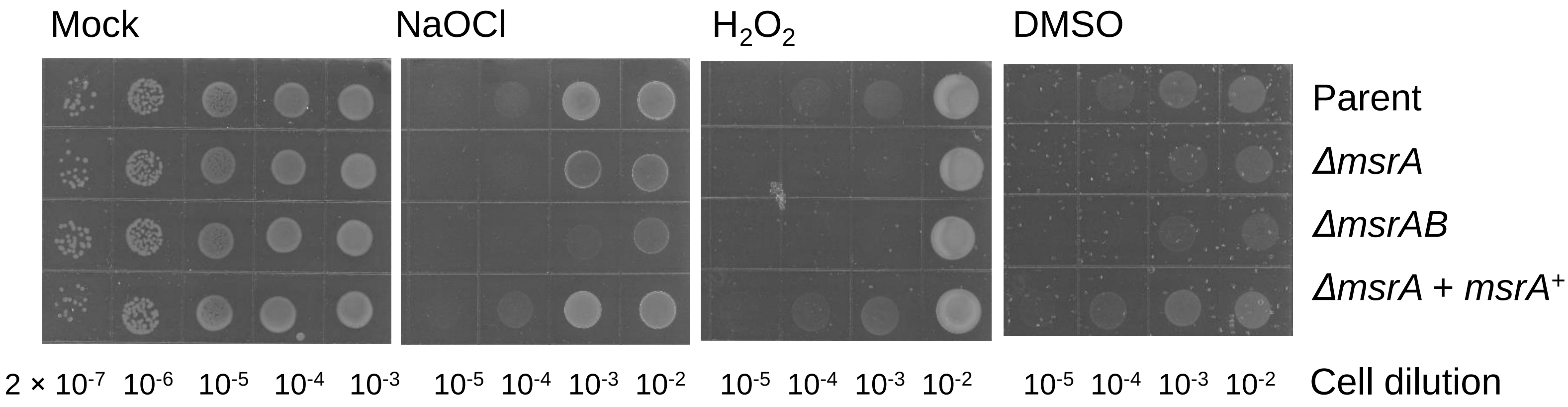

Supplemental Fig. S7. Hypersensitivity of *Hfx. volcanii* methionine sulfoxide reductase mutants to oxidative stress. Parent (H26), *ΔmsrA* (YW1005), *ΔmsrA ΔmsrB* (LR01) and *ΔmsrA* + *msrA*$^+$ (YW1005-pJAM3200) were serially diluted and spot plated onto glycerol minimal medium supplemented with mock control, NaOCl (0.5 mM), H_2O_2 (0.5 mM), and DMSO (1 M), as indicated. See methods for details.
