# Supplementary material for: Methionine Sulfoxide Reductase A (MsrA) and Its Function in Ubiquitin-Like Protein Modification in Archaea
Source: mBio. 2017 Sep 5;8(5):e01169-17. doi: 10.1128/mBio.01169-17 (PMC5587910; doi:10.1128/mBio.01169-17)
Supplement: FIG S8 [file mbo004173464sf8.pdf]

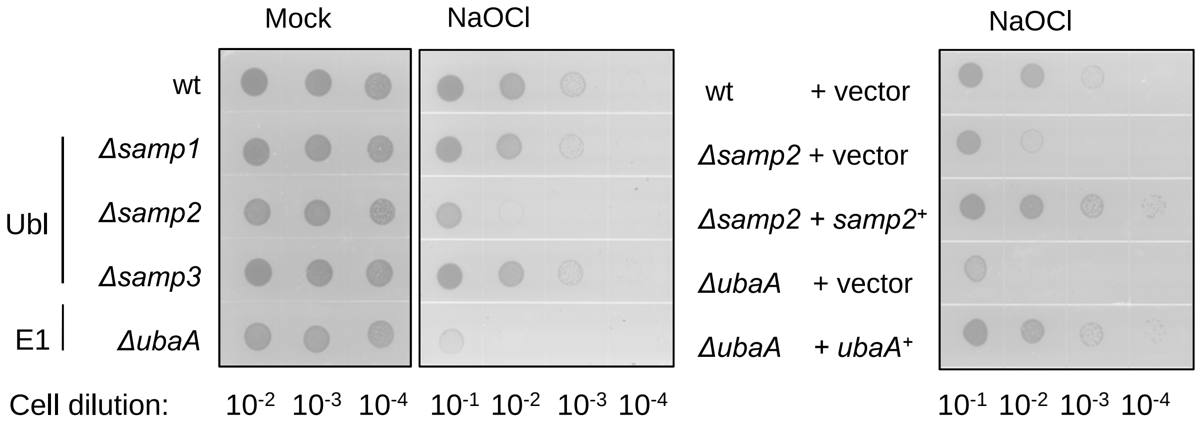

Supplemental Fig. S8. Hypersensitivity of *Hfx. volcanii* ubiquitin-like modification system mutants (*ΔubaA and Δsamp2*) to oxidative stress. *Hfx. volcanii* strains were grown to log-phase in ATCC974 medium and exposed to NaOCl (16 mM) or a mock control for 30 min, as indicated. Cells were examined for survival by spot serial dilution assay onto ATCC974 medium. The dilution of cells is indicated below each plate. Strains were parent (H26, wt), *Δsamp1* (HM1014)*, Δsamp2* (HM1042), *Δsamp3* (HM1055), *ΔubaA* (HM1052), *ΔubaA* + *ubaA*$^{+}$ (HM1052-pJAM957), *Δsamp2* + *samp2*$^{+}$ (HM1042-pJAM949), parent + vector (H26-pJAM202c), *Δsamp2* + vector (HM1042-pJAM202c), and *ΔubaA* + vector (HM1052-pJAM202c). wt, wild type. vector, empty vector control. See methods for details.
