# Supplementary material for: Methionine Sulfoxide Reductase A (MsrA) and Its Function in Ubiquitin-Like Protein Modification in Archaea
Source: mBio. 2017 Sep 5;8(5):e01169-17. doi: 10.1128/mBio.01169-17 (PMC5587910; doi:10.1128/mBio.01169-17)
Supplement: TABLE S1 [file mbo004173464st1.pdf]

Supplemental Table S1. List of strains and plasmids used in this study.

Strain, plasmid	Description[a]	Source or reference
**Strains:**		
E. coli		
Top10	F^- *recA1 endA1 hsdR17*(r_K^- m_K^+) *supE44 thi-1 gyrA relA1*	Invitrogen
Rosetta(DE3)	F^- *ompT hsdS*B(rB^-mB^-) *gal dcm* (DE3) pRARE (Cm^r)	Novagen
GM2163	F^- *ara-14 leuB6 fhuA31 lacY1 tsx78 glnV44 galK2 galT22 mcrA dcm-6 hisG4 rfbD1 rpsL136 dam13*::Tn*9 xylA5 mtl-1 thi-1 mcrB1 hsdR2*	New England Biolabs
XL10-Gold	Tet^r *Δ(mcrA)183 Δ(mcrCB-hsdSMR-mrr)173 endA1 supE44 thi-1 recA1 gyrA96 relA1 lac* Hte [F´ *proAB lacIqZΔM15 Tn10* (Tet^r) Amy Cm^r]	Agilent Technologies
Hfx. volcanii		
DS2	Dead sea isolate	(1)
DS70	DS2 cured of plasmid pHV2	(2)
H26	DS70 *ΔpyrE2*	(3)
HM1041	H26 *Δsamp1*	(4)
HM1042	H26 *Δsamp2*	(4)
HM1055	H26 *Δsamp3*	(4)
HM1052	H26 *ΔubaA*	(4)
NH02	H26 *Δsamp1Δsamp2Δsamp3ΔubaA*	This study
YW1005	H26 *ΔmsrA*	This study
YW1006	H26 *ΔmsrB*	This study
LR01	H26 *ΔmsrAΔmsrB*	This study
LR02	H26 *Δsamp1Δsamp2Δsamp3ΔmsrA*	This study
LR03	H26 *Δsamp1Δsamp2Δsamp3ΔmsrAΔubaA*	This study
XF124	H26 *Δsamp1Δsamp2Δsamp3ΔmsrAΔmoaE*	This study
**Plasmids:**		
pJAM202c	Ap^r; Nv^r; *Hfx. volcanii-E. coli* shuttle plasmid, empty vector	(5)
pJAM947	Ap^r; Nv^r; pJAM202c carries $P2_{rrnA}$:*flag-samp1*	(6)
pJAM556	Ap^r; Nv^r; pJAM202c carries $P2_{rrnA}$:*flag-samp1 S85R*	(7)
pJAM949	Ap^r; Nv^r; pJAM202c carries $P2_{rrnA}$:*flag-samp2*	(6)
pJAM957	Ap^r; Nv^r; pJAM202c carries $P2_{rrnA}$:*ubaA-strepII*	(4)
pJAM977	Ap^r; Nv^r; pJAM202c carries $P2_{rrnA}$:*flag-samp3*	(8)
pJAM3010	Ap^r; Nv^r; pJAM202c carries $P2_{rrnA}$:*msrA-strepII*	This study
pJAM3011	Ap^r; Nv^r; pJAM202c carries $P2_{rrnA}$:*msrB-strepII*	This study
pJAM3202	Ap^r; Nv^r; pJAM202c carries $P2_{rrnA}$:*flag-samp1* and *msrA-strepII*	This study
pJAM3203	Ap^r; Nv^r; pJAM202c carries $P2_{rrnA}$:*flag-samp2* and *msrA-strepII*	This study
pJAM3204	Ap^r; Nv^r; pJAM202c carries $P2_{rrnA}$:*flag-samp3* and *msrA-strepII*	This study
pJAM3212	Ap^r; Nv^r; pJAM202c carries $P2_{rrnA}$:*flag-samp1* and $msrA_{C13S}$-*strepII*	This study
pJAM3213	Ap^r; Nv^r; pJAM202c carries $P2_{rrnA}$:*flag-samp2* and $msrA_{C13S}$-*strepII*	This study
pJAM3214	Ap^r; Nv^r; pJAM202c carries $P2_{rrnA}$:*flag-samp3* and $msrA_{C13S}$-*strepII*	This study
pJAM2275	Ap^r; Nv^r; pJAM202c carries $P2_{rrnA}$:*flag-samp2* and $msrA_{C16S}$-*strepII*	This study
pJAM2276	Ap^r; Nv^r; pJAM202c carries $P2_{rrnA}$:*flag-samp2* and $msrA_{C48S}$-*strepII*	This study
pJAM2277	Ap^r; Nv^r; pJAM202c carries $P2_{rrnA}$:*flag-samp2* and $msrA_{C162S}$-*strepII*	This study
pJAM2283	Ap^r; Nv^r; pJAM202c carries $P2_{rrnA}$:*flag-samp2* and $msrA_{E56A}$-*strepII*	This study
pET24b	Km^r, IPTG induced expression plasmid	Novagen
pJAM3200	Km^r, pET24b carries *msrA-strepII*	This study
pJAM2273	Km^r, pET24b carries $msrA_{C13S}$-*strepII*	This study
pJAM2284	Km^r, pET24b carries $msrA_{E56A}$-*strepII*	This study
pTA131	Ap^r; Nv^r; *pyrE2*-based integration vector	(3)
pJAM3012	Ap^r; pTA131 carries *ΔmsrA* with 540-bp 5' and 3' flanking sequences	This study
pJAM3219	Ap^r; pTA131 carries *ΔmsrB* with 500-bp 5' and 3' flanking sequences	This study
pJAM1114	Ap^r; pTA131 carries *ΔmoaE* with 500-bp 5' and 3' flanking sequences	(4)

[a]Ap^r, ampicillin resistance; Nv^r, novobiocin resistance; Tet^r, tetracycline resistance; Cm^r, chloramphenicol resistance; Km^r, kanamycin resistance. SAMP1, HVO_2619; SAMP2, HVO_0202; SAMP3, HVO_2177; UbaA, HVO_0558; MoaE, HVO_1864; MsrA, HVO_A0230; MsrB, HVO_2234.

References - Supplementary Table S1

1. **Mullakhanbhai MF, Larsen H.** 1975. *Halobacterium volcanii* spec. nov., a Dead Sea halobacterium with a moderate salt requirement. Arch Microbiol **104:**207-214.
2. **Wendoloski D, Ferrer C, Dyall-Smith ML.** 2001. A new simvastatin (mevinolin)-resistance marker from *Haloarcula hispanica* and a new *Haloferax volcanii* strain cured of plasmid pHV2. Microbiology **147:**959-964.
3. **Allers T, Ngo HP, Mevarech M, Lloyd RG.** 2004. Development of additional selectable markers for the halophilic archaeon *Haloferax volcanii* based on the *leuB* and *trpA* genes. Appl Environ Microbiol **70:**943-953.
4. **Miranda H, Nembhard N, Su D, Hepowit N, Krause D, Pritz J, Phillips C, Söll D, Maupin-Furlow J.** 2011. E1- and ubiquitin-like proteins provide a direct link between protein conjugation and sulfur transfer in archaea. Proc Natl Acad Sci U S A **108:**4417-4422.
5. **Zhou G, Kowalczyk D, Humbard M, Rohatgi S, Maupin-Furlow J.** 2008. Proteasomal components required for cell growth and stress responses in the haloarchaeon *Haloferax volcanii*. J Bacteriol **190:**8096-8105.
6. **Humbard M, Miranda H, Lim J, Krause D, Pritz J, Zhou G, Chen S, Wells L, Maupin-Furlow J.** 2010. Ubiquitin-like small archaeal modifier proteins (SAMPs) in *Haloferax volcanii*. Nature **463:**54-60.
7. **Dantuluri S, Wu Y, Hepowit NL, Chen H, Chen S, Maupin-Furlow JA.** 2016. Proteome targets of ubiquitin-like samp1ylation are associated with sulfur metabolism and oxidative stress in *Haloferax volcanii*. Proteomics **16:**1100-1110.
8. **Miranda HV, Antelmann H, Hepowit N, Chavarria NE, Krause DJ, Pritz JR, Bäsell K, Becher D, Humbard MA, Brocchieri L, Maupin-Furlow JA.** 2014. Archaeal ubiquitin-like SAMP3 is isopeptide-linked to proteins via a UbaA-dependent mechanism. Mol Cell Proteomics **13:**220-239.
