# Supplementary material for: Methionine Sulfoxide Reductase A (MsrA) and Its Function in Ubiquitin-Like Protein Modification in Archaea
Source: mBio. 2017 Sep 5;8(5):e01169-17. doi: 10.1128/mBio.01169-17 (PMC5587910; doi:10.1128/mBio.01169-17)
Supplement: TABLE S2 [file mbo004173464st2.pdf]

Supplemental Table S2. List of primers used in this study.

Primer Pair[a]	Primer Sequence (5'-3')[b]	Description; construct generated
MsrA NdeI FW<br>MsrA KpnI RV	5'-aacatatgGGAAGCACACAGACTGCGACGTT-3'<br>5'-ttggtaccGGCGTCCGGCTCCGCCT-3'	PCR of *msrA*; pJAM3010
MsrA-540 BamHI FW<br>MsrA-540 HindIII FW	5'-taggatccCCGCGCCAACTGCCCGGTC-3'<br>5'-ataagcttGGCGCTGGACGACCTCCACTCC-3'	PCR of *msrA* and 540 bp flanking 5' and 3' of *msrA*; '*msrA* pre-deletion' plasmid
MsrA inverse FW<br>MsrA inverse RV	5'-CCCTGCTGGAGGCCGGTCCG-3'<br>5'-AGCCTCGGTTACGTCGCCGGG-3'	inverse PCR of '*msrA* pre-deletion' plasmid; pJAM3012
MsrA-700 FW<br>MsrA-700 RV	5'-GGCTCCAGAGAAGGCGGCGGGA-3'<br>5'-CGTTCCCGCTCGACGACGAACACCGC-3'	PCR confirm *ΔmsrA* strains, anneals 700 bp 5' and 3' of *msrA*
MsrB NdeI FW<br>MsrB KpnI RV	5'-aacatatgAGCGACAGCGAATTCAGCCTC-3'<br>5'-ttggtaccGTTCTCGTCCGCCTCGAAGTCG-3'	PCR of *msrB*; pJAM3011
MsrB-500 up FW<br>MsrB-500 up RV	5'-AATTCCTGCAGCCCGGGGGATCCTCTGAGGTAACAACAATGTAC-3'<br>5'-GTTCTGGTTCTGTCGGCGAGAAGGTTCGGACGC-3'	PCR 500 bp region 5' of *msrB*; pJAM3219 by SLIC
MsrB-500 down FW<br>MsrB-500 down RV	5'-GCGTCCGAACCTTCTC GCCGACAGAACCAGAAC-3'<br>5'-CGCTCTAGAACTAGTGGATCCGTCTCGCTCGCCAT-3'	PCR 500 bp region 3' of *msrB*; pJAM3219 by SLIC
MsrB-700 FW<br>MsrB-700 RV	5'-GCCCGAAGGGGAAACAC-3'<br>5'-CCGATGAACGCCAACCA-3'	PCR confirm *ΔmsrB* strains, anneals 700 bp 5' and 3' of *msrB*
MsrA C13S FW<br>MsrA C13S RV	5'-GACGTTCGGCGGCGGC***TCG***TTCTGGTGCGTCGAAG-3'<br>5'-CTTCGACGCACCAGAA***CGA***GCCGCCGCCGAACGTC-3'	site-directed mutagenesis; MsrA C13S
MsrA C16S FW<br>MsrA C16S RV	5'-GGCGGCGGCTGTTTCTGG***TCG***GTCGAAGCCGCGTTCAAG-3'<br>5'-CTTGAACGCGGCTTCGAC***CGA***CCAGAAACAGCCGCCGCC-3'	site-directed mutagenesis; MsrA C16S
MsrA C48S FW<br>MsrA C48S RV	5'-CCCGAGCTACGAGCAGGTC***TCG***TCGGGGAGCACCGGCCACG-3'<br>5'-CGTGGCCGGTGCTCCCCGA***CGA***GACCTGCTCGTAGCTCGGG-3'	site-directed mutagenesis; MsrA C48S
MsrA C162S FW<br>MsrA C162S RV	5'-CTAACGACGCCTAC***TCG***CAGTTCAACGTCGTCC -3'<br>5'-GGACGACGTTGAACTG***CGA***GTAGGCGTCGTTAG -3'	site-directed mutagenesis; MsrA C162S
MsrA E56A FW<br>MsrA E56A RV	5'-GAGCACCGGCCACGCC***GCG***GTCGTGCAGGTCGAC-3'<br>5'-GTCGACCTGCACGAC***CGC***GGCGTGGCCGGTGCTC-3'	site-directed mutagenesis; MsrA E56A
MoaE-700 FW<br>MoaE-700 RV	5'-CGCCGCGATGAGCAGGCG-3'<br>5'-AGTCGCGTCTCGGTTCGGTTTCCG-3'	PCR confirm *ΔmoaE* mutants, anneals 700 bp 5' and 3' of *moaE*

[a]MsrA, HVO_A0230; MsrB, HVO_2234; MoaE, HVO_1864. [b]Oligonucleotide sequences introduced in primers to facilitate cloning are in lowercase with sites for restriction enzyme cleavage underlined and sites for site-directed mutagenesis are italicized, underlined and in bold.
